# Supplementary material for: Coherent optical coupling to surface acoustic wave devices
Source: Nat Commun. 2024 May 11;15:3993. doi: 10.1038/s41467-024-48167-7 (PMC11088653; doi:10.1038/s41467-024-48167-7)
Supplement: Supplementary file 1 — Supplementary Information [file 41467_2024_48167_MOESM1_ESM.docx]

**Supplementary Information for:**

**Coherent Optical Coupling to Surface Acoustic Wave Devices**

Arjun Iyer^1,*^, Yadav P. Kandel^2^, Wendao Xu^1^, John M. Nichol^2^ and William H. Renninger^1,2^

**S1. Theoretical Estimation of the Optomechanical Coupling Strength**

In this Section, we derive an analytic expression for the coupling rate for parametric coupling between traveling-wave non-collinear optical fields and a standing wave SAW cavity mode. Minimal assumptions are made, enabling a useful alternative to computationally intensive FEM calculations. The result of this analysis is a simple analytical expression for the coupling rate as a function of material and design parameters.

The system is modeled as a semi-infinite crystalline medium occupying the region $z<0$ and supporting surface acoustic waves confined to the material interface, $z=0$ (Fig. S1). Periodic acoustic reflectors along the x-axis confine surface acoustic fields in a Gaussian SAW cavity. Two non-collinear optical fields, pump and Stokes, subtend equal but opposite angles ($\theta$) with the z-axis and are incident from outside the medium ($z>0$) on the region enclosed by the two acoustic mirrors. Because optomechanical scattering is a vectorial process, the resulting optomechanical coupling is a function of the polarization of the incident optical fields. Optomechanical coupling strengths are derived for the cases where, 1) both optical fields are TE polarized (TE-TE), 2) the pump is TE-polarized, and the Stokes field is TM-polarized (TE-TM), and 3) both fields are TM-polarized (TM-TM).


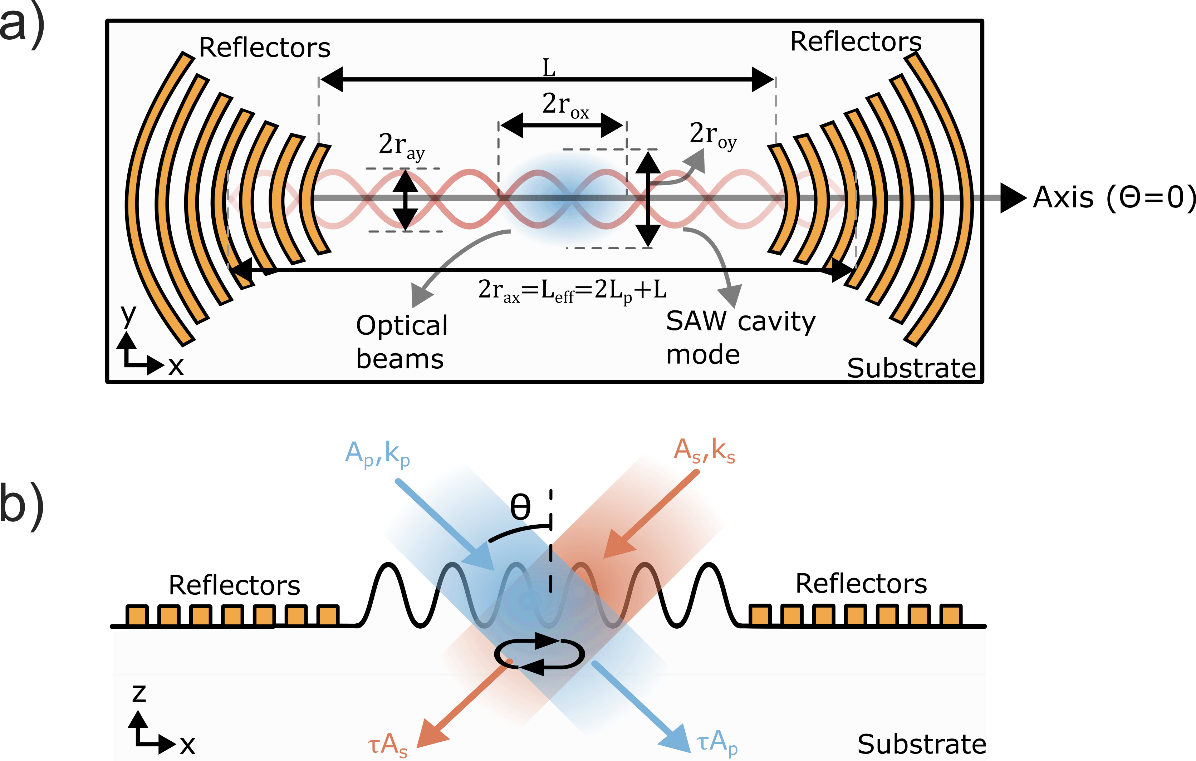


Figure S1: SAW based optomechanical device for calculating coupling rates. a) Top view (XY) of the optomechanical system with the relevant optical and acoustic dimensions. b) Side view (XZ) of the optomechanical device.

**A) TE-polarized Optical Fields**

In the case of TE-polarized optical fields, the electric field for the pump ($E_{p})$ and Stokes ($E_{s}$) fields outside the medium close to the surface ($z\to0^{+}$) are given as

$E_{p}\left( x,y, z=0^{+} \right)=A_{p}\exp\left( -\frac{x^{2}}{r_{ox}^{2}}-\frac{y^{2}}{r_{oy}^{2}} \right)\exp\left( {ik}_{p}\sin\theta x \right) \hat{y},$ ( 1 )

$E_{s}\left( x,y, z=0^{+} \right)=A_{s}\exp\left( -\frac{x^{2}}{r_{ox}^{2}}-\frac{y^{2}}{r_{oy}^{2}} \right)\exp\left( {-ik}_{s}\sin\theta x \right) \hat{y},$ ( 2 )

where $A_{p}(A_{s})$ and $k_{p}(k_{s})$ refer to the amplitude and the wavevector of the pump (Stokes) optical field, respectively. Since the acoustic frequencies of interest are much smaller than the optical frequencies, $k_{p}\approx k_{s}=k_{0}$. $r_{\mathrm{ox}}$ and $r_{\mathrm{oy}}$ refer to the effective optical beam radius along the x and the y-axis, respectively. Since the fields are incident at an angle, the resultant distribution on the surface is not symmetric along the x and y-axes. The effective beam waist along the x-axis ($r_{\mathrm{ox}}$) can be expressed as $r_{\mathrm{ox}}=r_{0}/\cos\theta$, while the beam waist along the y-axis remains unchanged as $r_{\mathrm{oy}}=r_{0}$, where $r_{0}$ is the incident beam radius, assumed to be the same for both the pump and Stokes fields. The electric fields on the other side ($z\to0^{-}$) of the interface can be derived from Eq. 1 and Eq. 2 by multiplying the appropriate Fresnel transmission coefficients ($\tau(\theta)$) as

$E_{p}\left( x,y, z=0^{-} \right)=A_{p}\tau(\theta)\exp\left( -\frac{x^{2}}{r_{\mathrm{ox}}^{2}}-\frac{y^{2}}{r_{\mathrm{oy}}^{2}} \right)\exp\left( \mathrm{ik}_{0}\sin\theta x \right) \hat{y},$ ( 3 )

$E_{s}\left( x,y, z=0^{-} \right)=A_{s}\tau(\theta)\exp\left( -\frac{x^{2}}{r_{\mathrm{ox}}^{2}}-\frac{y^{2}}{r_{\mathrm{oy}}^{2}} \right)\exp\left( {-ik}_{0}\sin\theta x \right) \hat{y}.$ ( 4 )

Accounting for the Gaussian mode profile of the m^th^ SAW cavity mode, the acoustic field can be expressed as [1,2]

$u_{x}=U_{0}\left( \exp\left( \eta q_{m}z-i\phi\right)+c.c. \right)\exp\left( -\frac{x^{2}}{r_{\mathrm{ax}}^{2}}-\frac{y^{2}}{r_{\mathrm{ay}}^{2}} \right)\cos(q_{m}x)$, ( 5 )

$u_{z}=-\frac{U_{0}}{i}\left( \gamma exp \left( \eta q_{m}z-i\phi\right)+c.c. \right)\exp\left( -\frac{x^{2}}{r_{\mathrm{ax}}^{2}}-\frac{y^{2}}{r_{\mathrm{ay}}^{2}} \right)\cos(q_{m}x)$, ( 6 )

where $u_{x}\mathrm{and}u_{z}$ are the x and the z-component of the acoustic displacement, respectively. $U_{0}, q_{m}, r_{\mathrm{ax}}$, and $r_{\mathrm{ay}}$ refer to the amplitude, the wavevector, and the waist of the cavity mode along the x-axis given by $r_{\mathrm{ax}}=L_{\mathrm{eff}}/2$, and the waist along the y-axis, respectively. Here, $L_{eff}$, is the effective acoustic cavity length. $\eta, \phi$, and $\gamma$ are material-dependent parameters obtained by solving the acoustic wave equation with appropriate boundary conditions [1,2]. ‘c.c’ refers to the complex conjugate of the term preceding it. Given that the acoustic displacements decay as $\sim\exp(Re \left( \eta\right)q_{m}z )$, the acoustic powers (${\sim|u_{x}|}^{2}{+|u_{z}|}^{2}$) would correspondingly decay as $\sim\exp(2Re \left( \eta\right)q_{m}z )$. Here, we define the acoustic decay length, $l_{a}$, as, $l_{a}=\frac{1}{2Re\left( \eta\right)q_{m}}$, which is typically of the order of one acoustic wavelength. The acoustic decay length can be understood as the characteristic length within which the energy of the surface acoustic field decays within the bulk of the substrate.

Given acoustic and electric fields, the traveling wave coupling rate ($g_{0}$) is defined from the conventional definition as [3]

$g_{0}=-\frac{\omega x_{\mathrm{zpf}}}{2}\frac{\left\langle f\cdot u \right\rangle}{N_{p}N_{s}}$, ( 7 )

where $\omega, f$, and $u$ refer to the optical frequency, the optical force distribution, and the acoustic field distribution, respectively. The acousto-optic overlap is defined by a vectorial overlap integral as $\left\langle f \cdot u \right\rangle=\int f{\cdot u}^{*}\mathrm{dV}$. The zero-point displacement of the mechanical mode, $x_{\mathrm{zpf}}$, is defined as $x_{\mathrm{zpf}}=\sqrt{\frac{\hbar}{2m_{\mathrm{eff}}\Omega_{m}}}$. Here, $\Omega_{m}$ refers to the frequency of the acoustic mode, $m_{\mathrm{eff}}=\int\rho\left( \left| u\cdot u^{*} \right|^{2} \right)\mathrm{dV}$ is the effective mass of the mechanical mode, and $\rho$ is the density of the substrate. $N_{p(s)}=\sqrt{{\frac{1}{2}\epsilon}_{0}\int\epsilon\left| E_{p(s)} \right|^{2}\mathrm{dV}}$ serve as optical power normalization factors. For the specific case of travelling optical fields, these volume integrals ($N_{p(s)}$) are performed over the effective interaction volume, which in the case of SAWs, is a mode with a Gaussian distribution on the surface penetrating an acoustic decay length, $l_{a}$, into the bulk of the material.

The traveling-wave coupling rate, as defined here, is similar in form to the coupling rate defined in conventional cavity optomechanics [3,4], except that the optical power normalizations ($N_{p}, N_{s}$) are only performed over the interaction volume and not over the entire optical cavity, as would be the case in standard cavity-optomechanical calculations [4,5]. In the case that the optical and acoustic cavities are not matched and have different sizes, the equivalent cavity optomechanical ($g_{0}^{c}$) coupling rate can be derived from $g_{0}$ as

$g_{0}^{c}=g_{0}\frac{N_{p}^{T}N_{s}^{T}}{N_{p}^{c}N_{s}^{c}}\approx g_{0}\frac{l_{a}}{l_{\mathrm{opt}}} ,$ ( 8 )

where $N_{p(s)}^{T(c)}$are the optical normalization factors for travelling-wave (cavity) pump (Stokes) fields. Assuming a constant transverse beam size, and small incident angles, this ratio of optical normalization factors can be simplified to ratio of the acoustic decay length, $l_{a}$, over which integrals are performed when deriving $g_{0}$, and optical cavity length, $l_{\mathrm{opt}}$, over which integrals would be performed in the case of optical cavities.

The strength of interactions studied in this work can also be quantified through a Brillouin-like gain coefficient ($G_{B}$) defined as [6]

$G_{B}=\frac{\omega Q_{m}}{2\Omega_{m}^{2}P_{p}P_{s}}\frac{\left| \left\langle f\cdot u \right\rangle\right|^{2}}{\langle u\cdot\rho u\rangle}$, ( 9 )

where $P_{p}\mathrm{and}P_{s}$ are the incident pump and Stokes powers, respectively. $Q_{m}$ is the mechanical quality factor. The travelling-wave coupling rate and the Brillouin gain coefficient are related as

$\left| g_{0} \right|=\left( \frac{v_{p}v_{s}\hbar\omega_{p}\Omega_{m}G_{B}}{4l_{a}^{2}Q_{m}} \right)^{\frac{1}{2}}.$ ( 10 )

While the coupling rate is typically used for optomechanical systems with an acoustic cavity, the Brillouin gain coefficient leads to a direct relationship for the efficiency of the optomechanical process. The Brillouin gain coefficient ($G_{B}$) can also be defined through

$\frac{\Delta P_{s}}{P_{s}}=G_{B}P_{p}$, ( 11 )

where $\Delta P_{s}$ refers to the change in Stokes power over the interaction volume and $P_{s}$ and $P_{p}$ refer to the input Stokes and pump power, respectively. In terms of $G_{B}$, the interactions demonstrated here are at least five orders of magnitude more efficient than previously measured optomechanical coupling to SAWs on planar devices [7]. In the next Sections, the acousto-optic contributions from the optical forces due to radiation pressure and electrostriction on the surface and the bulk of the medium are derived.

**Radiation Pressure**

The radiation pressure force, $P_{\mathrm{rp}},$ is given by [8]

$P_{\mathrm{rp}}=\frac{1}{2}\epsilon_{0} E_{\mathrm{pt}}\cdot E_{\mathrm{st}}^{*}\left( \epsilon-1 \right)-\frac{1}{2}\epsilon_{0}^{-1}D_{\mathrm{pn}}D_{\mathrm{sn}}^{*}\left( \frac{1}{\epsilon}-1 \right)$, ( 12 )

where $E_{p\left( s \right)t(n)}\mathrm{and}D_{p\left( s \right)t(n)}$ refer to the tangential (normal) pump (Stokes) electric and displacement fields, respectively. $\epsilon_{0} and \epsilon$refer to the dielectric permittivity of the vacuum and the relative permittivity material, respectively. Radiation pressure force points along the surface normal (along the positive z-axis). Note that, since there exist two tangential directions for a given surface normal, a vector dot product of the tangential fields is required for determining radiation pressure forces. For TE fields $E_{\mathrm{pz}}=E_{\mathrm{sz}}=0,$ and the resultant expression for $P_{\mathrm{rp}}$ can be simplified to

$P_{\mathrm{rp}}(x,y)=\frac{1}{2}\epsilon_{0}\left( \epsilon-1 \right)\left| \tau\right|^{2} A_{p} A_{s}^{*}e^{-2x^{2}/r_{\mathrm{ox}}^{2}} e^{-2y^{2}/r_{0}^{2}} e^{i{2k}_{0}\sin\theta x} \hat{z}$. ( 13 )

The resulting radiation pressure force only overlaps with the z-component of the acoustic displacement. The acoustic-optic overlap can be expressed as

$\left\langle f\cdot u \right\rangle_{\mathrm{rp}}=\int_{-\infty}^{\infty} dx dy P_{\mathrm{rp}}\left( x,y \right)u_{z}^{*}(z=0,x,y)$ ( 14 )

${=\epsilon}_{0}\frac{\epsilon-1}{2}\left| \tau\right|^{2} A_{p} A_{s}^{*} \frac{U_{0}}{i} 2Re\left( e^{-i\phi}\gamma\right) \int_{-\infty}^{\infty} \mathrm{dx}e^{-ax^{2}}e^{i\Delta kx}\cos q_{m}x \int_{-\infty}^{\infty} \mathrm{dy}e^{-by^{2}}$, ( 15 )

where we define additional parameters $a, \Delta k$, and $b$ as

$a=\frac{2}{r_{\mathrm{ox}}^{2}}+\frac{1}{r_{\mathrm{ax}}^{2}}$, ( 16 )

$b=\frac{2}{r_{0}^{2}}+\frac{1}{r_{\mathrm{ay}}^{2}}$, ( 17 )

$\Delta k=2k_{0}\sin\theta.$ ( 18 )

Since the integrals concerning the spatial variables $x$ and $y$ are independent, we separately calculate the two integrals

$I_{1}=\int_{-\infty}^{\infty} \mathrm{dy}e^{-by^{2}}=\sqrt{\frac{\pi}{b}}$ , ( 19 )

$I_{2}=\int_{-\infty}^{\infty} \mathrm{dx}e^{-ax^{2}}e^{i\Delta kx}\cos q_{m}x=\int_{-\infty}^{\infty} \mathrm{dx}e^{-ax^{2}}e^{i\Delta kx} \frac{1}{2}(e^{-iq_{m}x}+e^{+iq_{m}x} )=\frac{1}{2}e^{-\frac{\left( \Delta q \right)^{2}}{4a}}\sqrt{\frac{\pi}{a}}$ , ( 20 )

where $\Delta q=\Delta k-q_{m}$ is the wavevector difference between the optical forces and the SAW cavity mode. While deriving Eq. 20, the non-phase-matched term$\propto exp(i\left( \Delta k+q_{m} \right)x)$), is neglected, which is similar to the rotating wave approximation in atomic physics. Also, in Eq. 20, the resulting acousto-optic overlap is a function of phase-mismatch $\Delta q$ and is maximum when the optical forces are perfectly phase-matched with the acoustic cavity mode ($\Delta q=0$, i.e., $\Delta k=q_{m}$). This dependence results in the phase-matching bandwidth described in the main text. The next Section will explore further implications of acoustic overlap and coupling rate dependence on phase-mismatch. For the subsequent calculations in this Section, we assume $\Delta q=0$.

The acousto-optic overlap resulting from radiation pressure forces can now be expressed as

$\left\langle f\cdot u \right\rangle_{\mathrm{rp}}{=\epsilon}_{0}\frac{\epsilon-1}{2i}\left| \tau\right|^{2} {U_{0}A}_{p} A_{s}^{*}\mathrm{Re}\left( e^{-i\phi}\gamma\right)\frac{\pi}{\sqrt{\mathrm{ab}}} .$ ( 21 )

**Photoelastic Forces**

Time-varying electric fields within a dielectric material can generate time-varying photoelastic optical forces. Photoelastic stresses resulting in optical forces are derived from the photoelastic tensor. For a material with a cubic crystalline lattice whose principal axes are oriented along the assumed cartesian axis, the stress tensor in the Voigt notation is given as [8,9]

$\left( \begin{matrix} \sigma_{\mathrm{xx}} \\ \sigma_{\mathrm{yy}} \\ \sigma_{\mathrm{zz}} \\ \sigma_{\mathrm{zy}} \\ \sigma_{\mathrm{zx}} \\ \sigma_{\mathrm{xy}} \end{matrix} \right)=-\frac{1}{2}\epsilon_{0}n^{4}\left( \begin{matrix} p_{11} & p_{12} & p_{12} & 0 & 0 & 0 \\ p_{12} & p_{11} & p_{12} & 0 & 0 & 0 \\ p_{12} & p_{12} & p_{11} & 0 & 0 & 0 \\ 0 & 0 & 0 & p_{44} & 0 & 0 \\ 0 & 0 & 0 & 0 & p_{44} & 0 \\ 0 & 0 & 0 & 0 & 0 & p_{44} \end{matrix} \right)\left( \begin{matrix} E_{\mathrm{px}}E_{\mathrm{sx}}^{*} \\ E_{\mathrm{py}}E_{\mathrm{sy}}^{*} \\ E_{\mathrm{pz}}E_{\mathrm{sz}}^{*} \\ E_{\mathrm{pz}}E_{\mathrm{sy}}^{*}+E_{\mathrm{py}}E_{\mathrm{sz}}^{*} \\ E_{\mathrm{pz}}E_{\mathrm{sx}}^{*}+E_{\mathrm{px}}E_{\mathrm{sz}}^{*} \\ E_{\mathrm{py}}E_{\mathrm{sx}}^{*}+E_{\mathrm{px}}E_{\mathrm{sy}}^{*} \end{matrix} \right)$. ( 22 )

Here the crystal symmetry gives $p_{12}=p_{13}=p_{32}$, which might not be true if the coordinate system does not coincide with the principal crystal axes. The pump and Stokes electric fields are calculated within the material. For TE fields, $E_{\mathrm{px}}=E_{\mathrm{sx}}=E_{\mathrm{pz}}=E_{\mathrm{sz}}=0$ and the resultant stresses are

$\sigma_{\mathrm{xx}}={-\frac{1}{2}\epsilon_{0}n^{4}p}_{12}E_{\mathrm{py}}E_{\mathrm{sy}}^{*}$, ( 23 )

$\sigma_{\mathrm{yy}}={-\frac{1}{2}\epsilon_{0}n^{4}p}_{11}E_{\mathrm{py}}E_{\mathrm{sy}}^{*}$, ( 24 )

$\sigma_{\mathrm{zz}}={-\frac{1}{2}\epsilon_{0}n^{4}p}_{12}E_{\mathrm{py}}E_{\mathrm{sy}}^{*}$, ( 25 )

$\sigma_{\mathrm{xy}}=\sigma_{\mathrm{yz}}=\sigma_{\mathrm{zx}}=0$. ( 26 )

In a system comprising homogeneous materials, photoelastic forces can exist inside each material, resulting in body forces in the bulk of the medium and at material interfaces where discontinuous stresses are present, resulting in surface pressure (analogous to radiation pressure). We separately calculate the contribution to the acousto-optic overlap of photoelastic forces on the surface and within the bulk of the medium.

The excess photoelastic surface force on the interface ($z=0$) is given as

$P_{\mathrm{es}}=\sigma_{\mathrm{xz}}\left( z=0 \right) \hat{x}+\sigma_{\mathrm{yz}}\left( z=0 \right)\hat{y}+\sigma_{\mathrm{zz}}\left( z=0 \right)\hat{z}=-{\frac{1}{2}\epsilon_{0}n^{4}p}_{12}E_{\mathrm{py}}E_{\mathrm{sy}}^{*}\hat{z}$. ( 27 )

The acousto-optic overlap resulting from photoelastic surface pressure is given by

$\left\langle f\cdot u \right\rangle_{\mathrm{es}}=\int_{-\infty}^{\infty} dx dy P_{\mathrm{es}}\left( x,y \right)U_{z}^{*}(z=0,x,y),$ ( 28 )

${=-\frac{1}{2i}n^{4}p_{12}\epsilon}_{0}\left| \tau\right|^{2} {U_{0}A}_{p} A_{s}^{*}\mathrm{Re}\left( e^{-i\phi}\gamma\right)\frac{\pi}{\sqrt{\mathrm{ab}}} .$ ( 29 )

Next, the overlap resulting from bulk photoelastic forces is calculated. The photoelastic body forces can be determined from the divergence of stress components and its vectorial components are given as [9,10]

$f_{x}= -\partial_{x}\sigma_{\mathrm{xx}}-\partial_{y}\sigma_{\mathrm{xy}}-\partial_{z}\sigma_{\mathrm{xz}}= -\partial_{x}\sigma_{\mathrm{xx}}$, ( 30 )

$f_{y}= -\partial_{x}\sigma_{\mathrm{xy}}-\partial_{y}\sigma_{\mathrm{yy}}-\partial_{z}\sigma_{\mathrm{yz}} = -\partial_{y}\sigma_{\mathrm{yy}}$, ( 31 )

$f_{z}= -\partial_{x}\sigma_{\mathrm{xz}}-\partial_{y}\sigma_{\mathrm{zy}}-\partial_{z}\sigma_{\mathrm{zz}}= -\partial_{z}\sigma_{\mathrm{zz}}.$ ( 32 )

Since the SAW cavity modes of interest, Rayleigh SAWs, have no displacement along the y-axis, only $f_{x}$ and $f_{z}$ can contribute to the gain. The force along the z-axis, $f_{z}\propto\partial_{z}\left( E_{p}E_{s} \right)$, can be assumed to be negligible ($f_{z}\sim0)$because the optical fields diffract negligibly within the decay length of the SAW cavity mode ($\sim5 \mu m$), and therefore the gradient along the z-axis is nearly zero. Consequently, only the forces along the x-axis result in non-zero overlap with the SAW cavity mode.

Further expanding Eq. 30 gives

$f_{x}= -\frac{1}{2}\epsilon_{0} \epsilon^{2} p_{12}A_{p}A_{s0}^{*}\left| \tau\right|^{2}e^{-\frac{2x^{2}}{r_{\mathrm{ox}}^{2}}} e^{-\frac{2y^{2}}{r_{0}^{2}}} e^{i{2k}_{0}\sin\theta x}\left( -i\Delta k+\frac{4x}{r_{\mathrm{ox}}} \right)$. ( 33 )

The optomechanical overlap contribution from the bulk electrostricitve forces, assuming $\Delta k=q_{m}$, is then given by

$\left\langle f\cdot u \right\rangle_{\mathrm{eb}}=-\frac{1}{2}\epsilon_{0} \epsilon^{2} p_{12}A_{p}A_{s0}^{*}\left| \tau\right|^{2}U_{0}\int_{-\infty}^{\infty} \mathrm{dy}e^{-by^{2}}\int_{-\infty}^{0} \mathrm{dz}\left( \exp\left( \eta q_{m}z-i\phi\right)+ c.c. \right)^{*}\int_{-\infty}^{\infty} \mathrm{dx}\frac{1}{2}\left( -iq_{m}+\frac{4x}{r_{\mathrm{ox}}} \right) e^{i\Delta qx-ax^{2}}.$ ( 34 )

Eq. 34 can be further simplified by evaluating the integrals to

$\left\langle f\cdot u \right\rangle_{\mathrm{eb}}=-\frac{1}{2i}\epsilon_{0} \epsilon^{2} p_{12}A_{p}A_{s0}^{*}\left| \tau\right|^{2}U_{0}\mathrm{Re}\left( \frac{e^{-i\phi}}{\eta} \right)\frac{\pi}{\sqrt{\mathrm{ab}}}$. ( 35 )

The total optomechanical overlap can now be written as

$\left\langle f\cdot u \right\rangle_{\mathrm{tot}}=\left\langle f\cdot u \right\rangle_{\mathrm{rp}}+\left\langle f\cdot u \right\rangle_{\mathrm{es}}+\left\langle f\cdot u \right\rangle_{\mathrm{eb}}=\frac{i}{2}\epsilon_{0}A_{p}A_{s}^{*}\left| \tau\right|^{2}U_{0}\frac{\pi}{\sqrt{\mathrm{ab}}}\alpha^{\mathrm{TE}}$, ( 36 )

where $\alpha^{\mathrm{TE}}$ is defined as

$\alpha^{\mathrm{TE}}=\alpha_{\mathrm{rp}}^{TE}+\alpha_{es}^{TE}+\alpha_{eb}^{TE}=\left( -\left( \epsilon-1 \right)\mathrm{Re}\left( e^{-i\phi}\gamma\right)+p_{12}\epsilon^{2}\mathrm{Re}\left( e^{-i\phi}\gamma\right)+p_{12}\epsilon^{2}\mathrm{Re}\left( \frac{e^{-i\phi}}{\eta} \right) \right)$ , ( 37 )

for $\alpha_{rp}^{TE}=-\left( \epsilon-1 \right)\mathrm{Re}\left( e^{-i\phi}\gamma\right)$, $\alpha_{es}^{TE}=p_{12}\epsilon^{2}\mathrm{Re}\left( e^{-i\phi}\gamma\right)$, and $\alpha_{eb}^{TE}=p_{12}\epsilon^{2}\mathrm{Re}\left( \frac{e^{-i\phi}}{\eta} \right)$. The $\alpha$ parameters correspond to normalized acousto-optic overlap contributions for radiation pressure, surface electrostriction, and bulk electrostriction. The acoustic mode normalization is given by

$\langle u,\rho u\rangle=\int dV \rho(\left| u_{x} \right|^{2}+\left| u_{z} \right|^{2})$. ( 38 )

Inserting expressions for acoustic displacements from Eq. 5 and Eq. 6 into Eq. 38 and evaluating the integrals gives

$\langle u,\rho u\rangle=\left| U_{0} \right|^{2}\frac{\pi}{4q_{m}}r_{\mathrm{ay}}r_{\mathrm{ax}}\left( \frac{1}{\mathrm{Re}\left( \eta\right)}+Re\left( \frac{e^{-2i\phi}}{\eta} \right)+\frac{\left| \gamma\right|^{2}}{\mathrm{Re}\left( \eta\right)}+Re\left( \frac{\gamma^{2}}{\eta}e^{-i2\phi} \right) \right)$ . ( 39 )

Eq. 39 can be written more compactly as

$\langle u,\rho u\rangle=\left| U_{0} \right|^{2}\rho\frac{\pi}{4q_{m}}r_{\mathrm{ay}}r_{\mathrm{ax}}\delta$, ( 40 )

with $\delta$ defined as

$\delta=\frac{1}{\mathrm{Re}\left( \eta\right)}+Re\left( \frac{e^{-2i\phi}}{\eta} \right)+\frac{\left| \gamma\right|^{2}}{\mathrm{Re}\left( \eta\right)}+Re\left( \frac{\gamma^{2}}{\eta}e^{-i2\phi} \right)$. ( 41 )

Next, the pump and Stokes power normalization factors $N_{p}$ and $N_{s}$ are given by

$N_{p(s)}=\sqrt{\frac{1}{2}\epsilon_{0}\int\epsilon\left| E_{p(s)} \right|^{2}\mathrm{dV}}=\left( \frac{1}{2} \epsilon_{0}\epsilon{|A}_{p\left( s \right)}\left. \right|^{2} \left| \tau\right|^{2}\left( \frac{\pi r_{o}^{2}}{2} \right)l_{a} \right)^{\frac{1}{2}}$, ( 42 )

where $l_{a}$ is the acoustic decay length (Eq. 5,6).

Combining Eq. 7, Eq. 36, Eq. 40, and Eq. 42 the coupling rate for TE-TE scattering can be expressed as

$g_{0}^{TE-TE}=\frac{-i\omega\alpha^{\mathrm{TE}}}{\epsilon r_{o}^{2}l_{a}}\sqrt{\frac{2\hbar q_{m}}{r_{\mathrm{ay}}r_{\mathrm{ax}}\delta ab\rho\Omega_{m}\pi}}$, ( 43 )

and the Brillouin gain coefficient is given as

$G_{B, TE-TE}=\frac{8q_{m}\omega Q}{\Omega_{0}^{2}c^{2}\rho\pi}\times\frac{\left| \alpha^{\mathrm{TE}} \right|^{2}}{\epsilon\delta}\times\frac{1}{\mathrm{ab}r_{\mathrm{ay}}r_{\mathrm{ax}}r_{0}^{4}}$. ( 44 )

**B) TE-polarized Pump and TM-polarized Stokes Optical Fields**

When the optical fields are cross-polarized, assuming the pump field is TE-polarized, as in the previous case, the Stokes field will be TM-polarized. The electric fields inside the medium, close to the surface ($z\to0^{-}$) are given as

$E_{p}\left( x,y, z=0^{-} \right)=A_{p}\tau\exp\left( -\frac{x^{2}}{r_{\mathrm{ox}}^{2}}-\frac{y^{2}}{r_{0}^{2}} \right)\exp\left( \mathrm{ik}_{0}\sin\theta x \right) \hat{y}$, ( 45 )

$E_{s}\left( x,y, z=0^{-} \right)=A_{s}\tau(-\cos\theta\hat{x}+\sin\theta\hat{z})\exp\left( -\frac{x^{2}}{r_{\mathrm{ox}}^{2}}-\frac{y^{2}}{r_{0}^{2}} \right)\exp\left( {-ik}_{0}\sin\theta x \right)$. ( 46 )

Here the Fresnel transmission coefficients for TE and TM polarizations are assumed to be approximately equal, which is generally true for small angles of incidence.

**Radiation Pressure**

Since the pump and Stokes optical fields do not have overlapping non-zero electric field components (they are perpendicularly polarized), i.e. $E_{\mathrm{pt}}\cdot E_{\mathrm{st}}^{*}=D_{\mathrm{pn}}D_{\mathrm{sn}}^{*}=0$ the net radiation pressure is zero, and by extension, the acoustic overlap is zero:

$\left\langle f\cdot u \right\rangle_{\mathrm{rp}}=0$. ( 47 )

**Photoelastic Forces**

Photoelastic stresses for cross-polarized optical fields are given by

$\left( \begin{matrix} \sigma_{\mathrm{xx}} \\ \sigma_{\mathrm{yy}} \\ \sigma_{\mathrm{zz}} \\ \sigma_{\mathrm{zy}} \\ \sigma_{\mathrm{zx}} \\ \sigma_{\mathrm{xy}} \end{matrix} \right)=-\frac{1}{2}\epsilon_{0}n^{4}\left( \begin{matrix} p_{11} & p_{12} & p_{12} & 0 & 0 & 0 \\ p_{12} & p_{11} & p_{12} & 0 & 0 & 0 \\ p_{12} & p_{12} & p_{11} & 0 & 0 & 0 \\ 0 & 0 & 0 & p_{44} & 0 & 0 \\ 0 & 0 & 0 & 0 & p_{44} & 0 \\ 0 & 0 & 0 & 0 & 0 & p_{44} \end{matrix} \right)\left( \begin{matrix} E_{\mathrm{px}}E_{\mathrm{sx}}^{*} \\ E_{\mathrm{py}}E_{\mathrm{sy}}^{*} \\ E_{\mathrm{pz}}E_{\mathrm{sz}}^{*} \\ E_{\mathrm{pz}}E_{\mathrm{sy}}^{*}+E_{\mathrm{py}}E_{\mathrm{sz}}^{*} \\ E_{\mathrm{pz}}E_{\mathrm{sx}}^{*}+E_{\mathrm{px}}E_{\mathrm{sz}}^{*} \\ E_{\mathrm{py}}E_{\mathrm{sx}}^{*}+E_{\mathrm{px}}E_{\mathrm{sy}}^{*} \end{matrix} \right).$ ( 48 )

The resultant photoelastic stresses are given by

$\sigma_{\mathrm{xx}}=\sigma_{\mathrm{yy}}=\sigma_{\mathrm{zz}}=\sigma_{\mathrm{zx}}=0$, ( 49 )

$\sigma_{\mathrm{zy}}={-\frac{1}{2}\epsilon_{0}n^{4}p}_{44}E_{\mathrm{py}}E_{\mathrm{sz}}^{*}$, ( 50 )

$\sigma_{\mathrm{xy}}={-\frac{1}{2}\epsilon_{0}n^{4}p}_{44}E_{\mathrm{py}}E_{\mathrm{sx}}^{*}$. ( 51 )

The photoelastic surface force is given by

$P_{\mathrm{es}}={\frac{1}{2}\epsilon_{0}n^{4}p}_{44}E_{\mathrm{py}}E_{\mathrm{sz}}^{*} \hat{y}$. ( 52 )

Since SAW cavity modes of interest have no displacement component along the y-axis, the resultant acoustic-optic overlap resulting from electrostrictive surface forces is 0:

$\left\langle f\cdot u \right\rangle_{\mathrm{es}}=0$. ( 53 )

Vectorial components of the photoelastic body forces are

$f_{x}= -\partial_{x}\sigma_{\mathrm{xx}}-\partial_{y}\sigma_{\mathrm{xy}}-\partial_{z}\sigma_{\mathrm{xz}}= -\partial_{y}\sigma_{\mathrm{xy}}$, ( 54 )

$f_{y}= -\partial_{x}\sigma_{\mathrm{xy}}-\partial_{y}\sigma_{\mathrm{yy}}-\partial_{z}\sigma_{\mathrm{yz}} = -\partial_{x}\sigma_{\mathrm{xy}}$, ( 55 )

$f_{z}= -\partial_{x}\sigma_{\mathrm{xz}}-\partial_{y}\sigma_{\mathrm{zy}}-\partial_{z}\sigma_{\mathrm{zz}}= -\partial_{y}\sigma_{\mathrm{zy}}$. ( 56 )

As assumed in the previous case, only the x and z-component of the photoelastic forces have non-zero overlap with acoustic displacements, since the SAW cavity mode does not have displacements along the y-axis. The forces along the x and the z-axis can be further expanded as

$f_{x}=\frac{1}{2}\epsilon_{0} \epsilon^{2} p_{44}A_{p}A_{s0}^{*}\left| \tau\right|^{2}\cos\theta\left( -\frac{4y}{r_{0}^{2}} \right)e^{-\frac{2x^{2}}{r_{\mathrm{ox}}^{2}}} e^{-\frac{2y^{2}}{r_{0}^{2}}}$, ( 57 )

$f_{z}=\frac{1}{2}\epsilon_{0} \epsilon^{2}p_{44}A_{p}A_{s0}^{*}\left| \tau\right|^{2}\sin\theta\left( -\frac{4y}{r_{0}^{2}} \right)e^{-\frac{2x^{2}}{r_{\mathrm{ox}}^{2}}} e^{-\frac{2y^{2}}{r_{0}^{2}}}$. ( 58 )

The acousto-optic overlap corresponding to forces along the x- and z-axis can now be determined as

$\left\langle f_{x}\cdot u_{x} \right\rangle=\frac{1}{2}\epsilon_{0} \epsilon^{2} p_{44}A_{p}A_{s0}^{*}\left| \tau\right|^{2}\cos\theta U_{0}\int_{-\infty}^{\infty} \mathrm{dy}\left( \frac{4y}{r_{0}^{2}} \right) e^{-by^{2}}\int_{0}^{\infty} \mathrm{dz}\left( \exp\left( \eta q_{m}z-i\phi\right)+ c.c \right)^{*}\int_{-\infty}^{\infty} \mathrm{dx} e^{-ax^{2}}.$ ( 59 )

Since $\int_{-\infty}^{\infty} \mathrm{dy}\left( \frac{4y}{r_{0}^{2}} \right) e^{-by^{2}}=0$, the contribution resulting from the x-component of photoelastic forces is 0, ($\left\langle f_{x}\cdot u_{x} \right\rangle=0)$.

Similarly, the z-component of the photoelastic body force also yields no overlap, i.e., $\left\langle f_{z}\cdot u_{z} \right\rangle=0$.

As a consequence, the total bulk electrostriction overlap is 0:

$\left\langle f\cdot u \right\rangle_{\mathrm{eb}}=0$. ( 60 )

The total overlap and, consequently, the optomechanical coupling rate for this configuration is 0:

$g_{0}^{TE-TM}=0$. ( 61 )

The absence of optomechanical coupling for the TE-TM scattering is primarily a result of the assumed crystal symmetry (cubic) and the resulting symmetry in the photoelastic tensor. In crystal structures with reduced symmetry, such as crystalline quartz and LiNbO_3_, SAW-mediated optomechanical processes can couple orthogonal polarizations.

**C) TM-polarized Optical Fields**

For the case where both pump and Stokes fields are TM-polarized, the electric fields inside the medium close to the surface ($z\to0^{-}$) are given as

$E_{p}\left( x,y, z=0^{-} \right)=A_{p}\tau(-cos \theta\hat{x}-\sin\theta\hat{z})\exp\left( -\frac{x^{2}}{r_{\mathrm{ox}}^{2}}-\frac{y^{2}}{r_{0}^{2}} \right)\exp\left( ik_{0}\sin\theta x \right)$, ( 62 )

$E_{s}\left( x,y, z=0^{-} \right)=A_{s}\tau\left( -cos \theta\hat{x}+\sin\theta\hat{z} \right)\exp\left( -\frac{x^{2}}{r_{\mathrm{ox}}^{2}}-\frac{y^{2}}{r_{0}^{2}} \right)\exp\left( {-ik}_{0}\sin\theta x \right)$. ( 63 )

**Radiation Pressure**

Radiation pressure forces, similar to cases A and B can be expressed as

$P_{\mathrm{rp}}=\frac{1}{2}\epsilon_{0}\left( \epsilon-1 \right)A_{p}A_{s}^{*}\left| \tau\right|^{2}\exp\left( -\frac{{2x}^{2}}{r_{\mathrm{ox}}^{2}}-\frac{{2y}^{2}}{r_{0}^{2}} \right) e^{i{2k}_{0}\sin\theta x}\left( \cos^{2} \theta-\epsilon\sin^{2} \theta\right) \hat{z}.$ ( 64 )

The corresponding acousto-optic overlap is calculated similarly to Eq. 15 giving

$\left\langle f\cdot u \right\rangle_{\mathrm{rp}}{=\epsilon}_{0}\frac{(\epsilon-1)(\cos^{2} \theta-\epsilon\sin^{2} \theta)}{2i}\left| \tau\right|^{2} {U_{0}A}_{p} A_{s}^{*}\mathrm{Re}\left( e^{-i\phi}\gamma\right)\frac{\pi}{\sqrt{\mathrm{ab}}}$. ( 65 )

**Photoelastic Forces**

The components of the photoelastic stress tensor are

$\sigma_{\mathrm{xx}}=-\frac{1}{2}\epsilon_{0}n^{4}p_{11}E_{\mathrm{px}}E_{\mathrm{sx}}^{*}-\frac{1}{2}\epsilon_{0}n^{4}p_{12}E_{\mathrm{pz}}E_{\mathrm{sz}}^{*}$, ( 66 )

$\sigma_{\mathrm{yy}}=0$, ( 67 )

$\sigma_{\mathrm{zz}}=-\frac{1}{2}\epsilon_{0}n^{4}p_{12}E_{\mathrm{px}}E_{\mathrm{sx}}^{*}-\frac{1}{2}\epsilon_{0}n^{4}p_{11}E_{\mathrm{pz}}E_{\mathrm{sz}}^{*}$, ( 68 )

$\sigma_{\mathrm{xz}}=-\frac{1}{2}\epsilon_{0}n^{4}p_{44}\left( E_{\mathrm{pz}}E_{\mathrm{sx}}^{*}+E_{\mathrm{px}}E_{\mathrm{sz}}^{*} \right)=0$, ( 69 )

$\sigma_{\mathrm{xy}}=\sigma_{\mathrm{yz}}=0$. ( 70 )

The electrostriction surface force on the interface is given by

$P_{\mathrm{es}}=-\frac{1}{2}\epsilon_{0}n^{4}A_{p}A_{s}^{*}\left| \tau\right|^{2}\left( p_{12}\cos^{2} \theta-p_{11}\sin^{2} \theta\right) \hat{z}$. ( 71 )

The resulting overlap with the acoustic mode is given as

$\left\langle f\cdot u \right\rangle_{\mathrm{es}}{=\frac{-1}{2i}n^{4}\left( p_{12}\cos^{2} \theta-p_{11}\sin^{2} \theta\right)\epsilon}_{0}\left| \tau\right|^{2} {U_{0}A}_{p} A_{s}^{*}\mathrm{Re}\left( e^{-i\phi}\gamma\right)\frac{\pi}{\sqrt{\mathrm{ab}}}$. ( 72 )

The electrostrictive body forces in the bulk of the substrate are given by

$f_{x}= -\partial_{x}\sigma_{\mathrm{xx}}-\partial_{y}\sigma_{\mathrm{xy}}-\partial_{z}\sigma_{\mathrm{xz}}= -\partial_{x}\sigma_{\mathrm{xx}}$, ( 73 )

$f_{y}= -\partial_{x}\sigma_{\mathrm{xy}}-\partial_{y}\sigma_{\mathrm{yy}}-\partial_{z}\sigma_{\mathrm{yz}} = 0$, ( 74 )

$f_{z}= -\partial_{x}\sigma_{\mathrm{xz}}-\partial_{y}\sigma_{\mathrm{zy}}-\partial_{z}\sigma_{\mathrm{zz}}= -\partial_{z}\sigma_{\mathrm{zz}}$. ( 75 )

Since the fields do not diffract significantly over the interaction volume, the z-component of the body force, $f_{z}$ is assumed to be zero ($\partial_{z}\sigma_{\mathrm{zz}}\sim0$), following reasoning from case A (TE-TE scattering). The resulting overlap is expressed as

$\left\langle f\cdot u \right\rangle_{\mathrm{es}}=\frac{i}{2}\epsilon_{0}\epsilon^{2}{(p}_{11}\cos^{2} \theta-p_{12}\sin^{2} \theta)A_{p}A_{s0}^{*}\left| \tau\right|^{2}U_{0}\mathrm{Re}\left( \frac{e^{i\phi}}{\eta} \right)\frac{\pi}{\sqrt{\mathrm{ab}}}$. ( 76 )

The total overlap is then given as

$\left\langle f\cdot u^{*} \right\rangle_{\mathrm{tot}}=\frac{i}{2}\epsilon_{0}A_{p}A_{s0}^{*}\left| \tau\right|^{2}U_{0}\frac{\pi}{\sqrt{\mathrm{ab}}}\left( \left( \epsilon-1 \right)\left( \cos^{2} \theta-\epsilon\sin^{2} \theta\right)\mathrm{Re}\left( e^{-i\phi}\gamma\right)-\left( p_{12}\cos^{2} \theta- p_{11}\sin^{2} \theta\right) \epsilon^{2}\mathrm{Re}\left( e^{-i\phi}\gamma\right)-{(p}_{12}\cos^{2} \theta-p_{11}\sin^{2} \theta)\epsilon^{2}\mathrm{Re}\left( \frac{e^{i\phi}}{\eta} \right) \right)$. ( 77 )

The optomechanical coupling rate for the TM-TM scattering process is given by

$g_{0}^{TM-TM}=\frac{-i\omega\alpha^{TM}}{\epsilon r_{o}^{2}l_{a}}\sqrt{\frac{2\hbar q_{m}}{r_{\mathrm{ay}}r_{\mathrm{ax}}\delta ab\rho\Omega_{m}\pi}}$, ( 78 )

where $\alpha^{\mathrm{TM}}$ is defined as

$$\alpha^{\mathrm{TM}}=\alpha_{\mathrm{rp}}^{TE}+\alpha_{es}^{TE}+\alpha_{eb}^{TE}$$

$=\left( \left( \epsilon-1 \right)\left( \cos^{2} \theta-\epsilon\sin^{2} \theta\right)\mathrm{Re}\left( e^{-i\phi}\gamma\right)-\epsilon^{2}\left( p_{12}\cos^{2} \theta-p_{11}\sin^{2} \theta\right)\mathrm{Re}\left( e^{-i\phi}\gamma\right)-\epsilon^{2}\left( p_{11}\cos^{2} \theta-p_{12}\sin^{2} \theta\right)\mathrm{Re}\left( \frac{e^{i\phi}}{\eta} \right) \right)$. ( 79 )

The corresponding Brillouin Gain coefficient is given by

$G_{B, TM-TM}=\frac{8q_{m}\omega Q}{\Omega_{0}^{2}c^{2}\rho\pi}\times\frac{\left| \alpha_{\mathrm{TM}} \right|^{2}}{\epsilon\delta}\times\frac{1}{\mathrm{ab}r_{\mathrm{ay}}r_{\mathrm{ax}}r_{0}^{4}}$. ( 80 )

Note that the strength of the TM-TM scattering process strongly depends on the optical angle of incidence and at larger angles it can be significantly stronger than the TE-TE scattering process.

The following material parameter values are used for [100]-cut GaAs to calculate coupling rates quoted in the main text.

| Parameter | Value | Description |
| --- | --- | --- |
| $\lambda_{o}$ | $1550.05 nm$ | Optical wavelength |
| $n$ | $3.37$ | Refractive index |
| $r_{0}$ | $30 \mu m$ | Optical beam radius |
| $\omega/2\pi=c/\lambda_{o}$ | $193.55 THz$ | Optical frequency |
| $p_{11}$ | $-0.165$ | Photoelastic constant |
| $p_{12}$ | $-0.140$ | Photoelastic constant |
| $\rho$ | $5307 kg m^{-3}$ | Density |
| $\theta$ | ${7.8}^{\circ}$ | The optical angle of incidence |
| $\theta_{m}=\theta$ | ${7.8}^{\circ}$ | Phase-matched angle corresponding to the SAW mode |
| $\lambda_{a}$ | $5.7 \mu m$ | Acoustic wavelength |
| $L$ | $505 \mu m$ | Acoustic mirror separation |
| $L_{p}$ | $7\lambda_{a}$ | SAW penetration depth |
| $L_{\mathrm{eff}}=L+2L_{p}$ | $620 \mu m$ | Effective SAW cavity length |
| $r_{\mathrm{ax}}=L/2$ | $292.6 \mu m$ | The effective radius of SAW mode along the x-axis |
| $r_{0a}$ | $4\lambda_{a}=24 \mu m$ | Acoustic waist radius along the y-axis |

**[110]-oriented Cavities**

Table S1: Physical parameters of [100]-cut GaAs used for calculating optomechanical coupling strengths

The acoustic parameters characterizing SAWs along [110]-direction on [100]-cut GaAs are [1]

| Parameter | Value | Description |
| --- | --- | --- |
| $v_{R}$ | $2865 m/s$ | Rayleigh SAW velocity |
| $\eta$ | $0.5+0.48i$ | SAW decay parameter |
| $\gamma$ | $0.68-1.16i$ | Parameter quantifying the ratio of SAW displacements along the x and z-axis |
| $\phi$ | $1.05$ | Phase lag between x and z-components of acoustic displacement |
| $Q$ | $7000$ | Acoustic quality factor |
| $\alpha^{TE} (\alpha^{TM})$ | $22.6 (24.1)$ | Overlap parameter for TE-TE (TM-TM) Scattering |
| $\alpha_{rp}^{TE}(\alpha_{rp}^{TM})$ | $7 (5.3)$ | Overlap contribution for radiation pressure forces for TE-TE (TM-TM) scattering |
| $\alpha_{es}^{TE}(\alpha_{es}^{TM})$ | $12.1 (11.6)$ | Overlap contribution for surface electrostriction forces for TE-TE (TM-TM) scattering |
| $\alpha_{eb}^{TE}(\alpha_{es}^{TM})$ | $3.5 (7.2)$ | Overlap contribution for bulk electrostriction forces for TE-TE (TM-TM) scattering |

Since the SAW direction of propagation is not along a principal crystalline axis, the photoelasticity tensor will have to be rotationally transformed, and as a result, $p_{12}\neq p_{13}$. The transformed coefficients of interest are $p_{12}=-0.078$and $p_{13}=-0.140$. The coupling rate and Brillouin gain coefficient for the TE-TE scattering process within [110]-oriented cavities is calculated as $\frac{g_{0}}{2\pi}=1.8\times{10}^{3}\mathrm{Hz}$ and $G_{B}=9\times{10}^{-7} W^{-1}$, respectively. For small angles, the coupling rate for TE-TE scattering is approximately equal to that for the TM-TM scattering process.

Table S2: SAW parameters assumed to calculate optomechanical gain coefficients for cavities oriented along the [110]-direction.

**[100]-oriented Cavities**

The acoustic parameters characterizing SAWs along [100]-direction on [100]-cut GaAs are [2]

| Parameter | Value | Description |
| --- | --- | --- |
| $v_{R}$ | $2615 m/s$ | Rayleigh SAW velocity |
| $\eta$ | $0.40+0.56i$ | SAW decay parameter |
| $\gamma$ | $0.37-1.1i$ | Parameter quantifying the ratio of SAW displacements along the x and z-axis |
| $\phi$ | $0.95$ | Phase lag between x and z-components of acoustic displacement |
| $Q$ | $120,000$ | Acoustic quality factor |
| $\alpha^{TE} (\alpha^{TM})$ | $27.6 (26.7)$ | Overlap parameter for TE-TE (TM-TM) scattering |
| $\alpha_{rp}^{TE}(\alpha_{rp}^{TM})$ | $7 (5.4)$ | Overlap contribution for radiation pressure forces for TE-TE (TM-TM) scattering |
| $\alpha_{es}^{TE}(\alpha_{es}^{TM})$ | $12.2 (11.7)$ | Overlap contribution for surface electrostriction forces for TE-TE (TM-TM) scattering |
| $\alpha_{eb}^{TE}(\alpha_{es}^{TM})$ | $8.4 (9.6)$ | Overlap contribution for bulk electrostriction forces for TE-TE (TM-TM) scattering |

The resultant TE-TE coupling rate and Brillouin gain coefficient for [100]-oriented cavities are calculated as $\frac{g_{0}}{2\pi}=1.73\times{10}^{3}\mathrm{Hz}$ and $G_{B}=2.5\times{10}^{-5} W^{-1}$, respectively.

Table S3: SAW parameters assumed to calculate optomechanical gain coefficients for cavities oriented along the [100]-direction.

The optomechanical coupling strength is a coherent interference of acousto-optic overlaps resulting from the three optical forces namely, radiation pressure, surface electrostriction, and bulk electrostriction, as revealed by Eq. 37 (or Eq. 79). In materials such as GaAs, which have relatively large photoelastic constants and large refractive indices, the three contributions are comparable (see values of $\alpha_{rp}, \alpha_{es},$ and $\alpha_{eb}$ in Table. S2 and S3). In other materials such as Si, which have large optical indices but weak photoelasticity, optomechanical interactions can be expected to be primarily dominated by radiation pressure. Inversely, in materials such as quartz, which support strong photoelasticity but have smaller optical indices, optomechanical interactions will be dominated by electrostrictive effects.

**S2. Phase Matching Envelope**

When calculating the acousto-optic overlaps in Section 2 (e.g., Eq. 20), optical fields were assumed to be perfectly phase-matched to acoustic modes $\Delta k=q_{m}$. In the absence of this assumption, the dependence of the optomechanical coupling rate on phase mismatch can be expressed as

$g_{0}\propto\left\langle f\cdot u \right\rangle\propto e^{-\frac{\left( \Delta q \right)^{2}}{4a}}$, ( 81 )

where $\Delta q=q_{m}-\Delta k$ quantifies the phase mismatch, and the parameter $a$ is defined in Eq. 16. Eq. 81 describes a Gaussian dependence of coupling rate on phase mismatch with a characteristic width quantified by the parameter, $\delta k=2\sqrt{a}=2\left( \frac{2}{r_{\mathrm{ox}}^{2}}+\frac{1}{r_{\mathrm{ax}}^{2}} \right)^{1/2}$. For experimental cavities investigated in this work, the acoustic cavity length ($r_{\mathrm{ax}}$) is much larger than the optical beam sizes (i.e. $r_{x}\ll r_{\mathrm{ax}}$ ), and as a result, $\delta k=2\sqrt{a}\approx\frac{2\sqrt{2}}{r_{\mathrm{ox}}}=\frac{2\sqrt{2}\cos\theta}{r_{0}}$. For small $\theta$, $\cos\theta\approx1$ and $\delta k\approx2\sqrt{2}/r_{0}$. As detailed in the main text, for small angles, the coupling rate has a Gaussian dependence on phase mismatch, with a characteristic width given by the inverse of the optical beam size.

The dependence of the coupling rate on the angle of incidence can be derived by expanding the phase mismatch as

$\Delta q=q_{m}-2k_{0}\sin\theta=2k_{0}\left( \sin\theta_{m}-\sin\theta\right),$ ( 82 )

where the phase-matching angle $\theta_{m}$ is defined as

$\theta_{m}=\sin^{-1} \left( \frac{q_{m}}{2k_{0}} \right)$. ( 83 )

For small angles of incidence $\theta$, $\sin\theta\approx\theta$, and the phase mismatch can be approximated as

$\Delta q\approx2k_{0}\left( \theta_{m}-\theta\right).$ ( 84 )

The dependence of the coupling rate can now be expressed as

$g_{0}\propto\exp\left( -\frac{\left( \theta-\theta_{m} \right)^{2}}{\delta\theta^{2}} \right)$, ( 85 )

where $\delta\theta=\frac{2\sqrt{a}}{2k_{0}}=\frac{\sqrt{2}}{k_{0}r_{0}}$.

For optical fields with free-space wavelength of $\lambda_{0}=1550 nm$, and the optical beam size of $r_{0}=30 \mu m$, the angular bandwidth is $\delta\theta={0.66}^{\circ}$, corresponding to a full width of $2\delta\theta={1.32}^{\circ},$ or the full-width-half-max (FWHM) ($\delta\theta_{HM}$) can be calculated as $\delta\theta_{HM}\approx{1.1}^{\circ}$. Since the Brillouin gain coefficient, $G_{B}$, varies as the square of the coupling rate, $G_{B}\propto g_{0}^{2}$, the corresponding angular bandwidth is given by $\delta\theta_{G}=\frac{1}{k_{0}r_{0}}={0.47}^{\circ}$ with a FWHM of $2\delta\theta_{G,HM}={0.78}^{\circ}$, which agrees well with the experimental results detailed in the main text.

**S3. Design and Simulation of Gaussian SAW cavities**

Gaussian SAW cavities in this work are based on a Fabry-Perot cavity design where two acoustic Bragg mirrors consisting of several metallic strips, confine surface acoustic modes between them. Each metallic strip reflects a small portion of the incident acoustic field, and the cumulative interference of all the reflectors achieves the desired acoustic confinement. As with optical Bragg gratings, the acoustic grating mirrors described here only efficiently confine SAW cavity modes whose frequencies are within the mirror stopbands. Interestingly, the optomechanical interactions described in this work, in addition to optically coupling to acoustic modes within the primary stop band can in principle optically couple SAW modes within other mirror stopbands as well. These modes, however, typically have lower acoustic quality factors. The geometry of the cavity is specified by four independent parameters: the direction of the cavity axis relative to a principal crystal axis (along the x-axis in Fig. S2a), the acoustic wavelength ($\lambda_{a})$, the acoustic beam waist at the center of the cavity ($w_{a}$), and the mirror separation ($L$) (Fig. S2a). For the chosen axis, the acoustic group velocity ($v_{g}(\Theta)$) is calculated as a function of the angle relative to the axis ($\Theta$) by numerically solving acoustic wave equations with appropriate boundary conditions [11]. Accounting for this anisotropy of the SAW velocity of the underlying substrate is essential to designing an efficient SAW cavity. For a Gaussian beam with a beam waist, $w_{a}$, and wavelength, $\lambda_{a}$, the acoustic Rayleigh range, $x_{aR}$, is given by $x_{aR}=\pi w_{a}^{2}/2\lambda_{a}$ and the corresponding phase along the propagation axis (x-axis) can be expressed as $\Phi\left( x \right)=k_{a}x+\frac{1}{2}\tan^{-1} (x/x_{aR}).$The first and second terms refer to the propagation and Guoy phases, respectively. The locations of the reflectors along the acoustic axis, $x_{i}$, can now be determined by calculating the nodes of the acoustic displacement, i.e., $\Phi\left( x_{i} \right)=n\pi$. The location of the reflector closest to the center, $x_{1}$, is chosen such that the separation between the first reflector of the two mirrors is approximately the chosen mirror separation, i.e. $x_{1}\approx L/2$. $L$ is chosen to be large enough to accommodate the optical beams incident on the device with minimal optical overlap with the acoustic mirrors. For efficient confinement of the acoustic field, the curvature of each metallic reflector must coincide with the local phase front of the desired Gaussian SAW mode. Ignoring the anisotropy in SAW velocity, the local phase front of a Gaussian beam at any reflector location $x_{i}$ would be circular arcs with a radius of curvature given by $R\left( x_{i} \right)=x_{i}\left( 1+\left( \frac{x_{aR}}{x_{i}} \right)^{2} \right)$. To account for the anisotropy of the substrate, a correction factor [12], $v_{g}(\Theta)/v_{g}\left( 0 \right)$, is introduced to obtain an angle-dependent radius-of-curvature function, $R^{'}\left( x_{i},\Theta\right)=R\left( x_{i} \right)v_{g}(\Theta)/v_{g}(0)$.

Figure S2: 3D FEM simulations of the designed SAW cavities. a) Cross-sectional (XY) illustration of Gaussian SAW cavities. The two acoustic mirrors consisting of numerous Al reflectors, confine a standing wave SAW mode along the x-axis with a Gaussian profile along the y-axis. The illustration also displays independent design parameters that characterize the cavity, namely, the acoustic wavelength ($\lambda_{a}$), cavity orientation relative to the crystallographic axes (x-axis), acoustic waist ($w_{a}$), and mirror separation ($L$). b) Example of a COMSOL simulation of the the fundamental Gaussian mode of a SAW cavity along [100]-directions on [100]-cut GaAs. Parameters of the simulation are $\lambda_{a}=5.7 \mu m$, $f_{a}\sim465 MHz$, $L\sim100 \mu m$, $w_{a}=3\lambda_{a}$ and reflector thickness $t=0.035\lambda_{a}$.


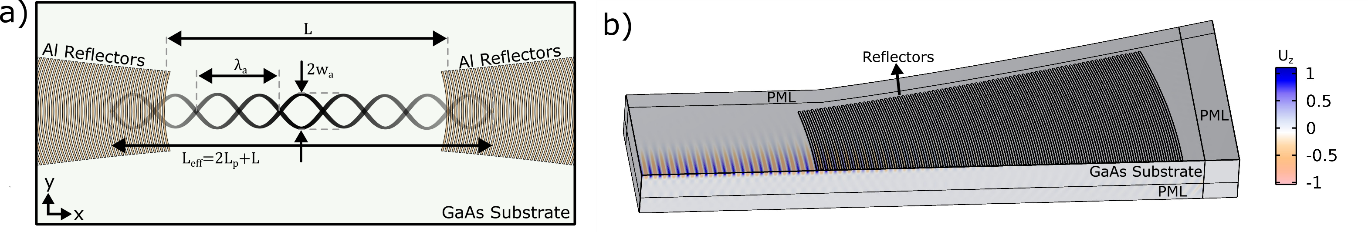


To validate the design principles, we perform 3D numerical finite element simulations (COMSOL 5.6) of SAW cavities on [100]-cut GaAs oriented along [100]-direction with $\lambda_{a}=5.7 \mu m,$ $L\sim100 \mu m$, and $w_{0}=3\lambda_{a}$. The thickness of the metallic reflectors is specified as a fraction of the acoustic wavelength and is set to be $\frac{t}{\lambda_{a}}=0.035$. This thickness has been reported to be a good balance between achieving tight confinement and achieving small acoustic mode volumes (thick electrodes), and mitigating acoustic scattering into the bulk of the substrate (thin electrodes) [13,14]. To minimize computational resources, we leverage the symmetry in our devices and simulate one-fourth of the entire device. The FEM geometry of the device (Fig. S2b) consisting of a substrate with a thickness of $3\lambda_{a}$ is surrounded by phase-matched layers with a thickness of $2\lambda_{a}$. In all areas of the device, the mesh size is ensured to be less than or equal to $\lambda_{a}/4$. Simulated devices have 50 metallic reflectors, which is reduced from 200 in the fabricated devices to limit computational resources required for the simulation. To accommodate the large number of degrees of freedom, the simulations are run on a supercomputing cluster with 56 nodes and 500-1000 GB RAM at the University of Rochester.

**S4. Device Fabrication**


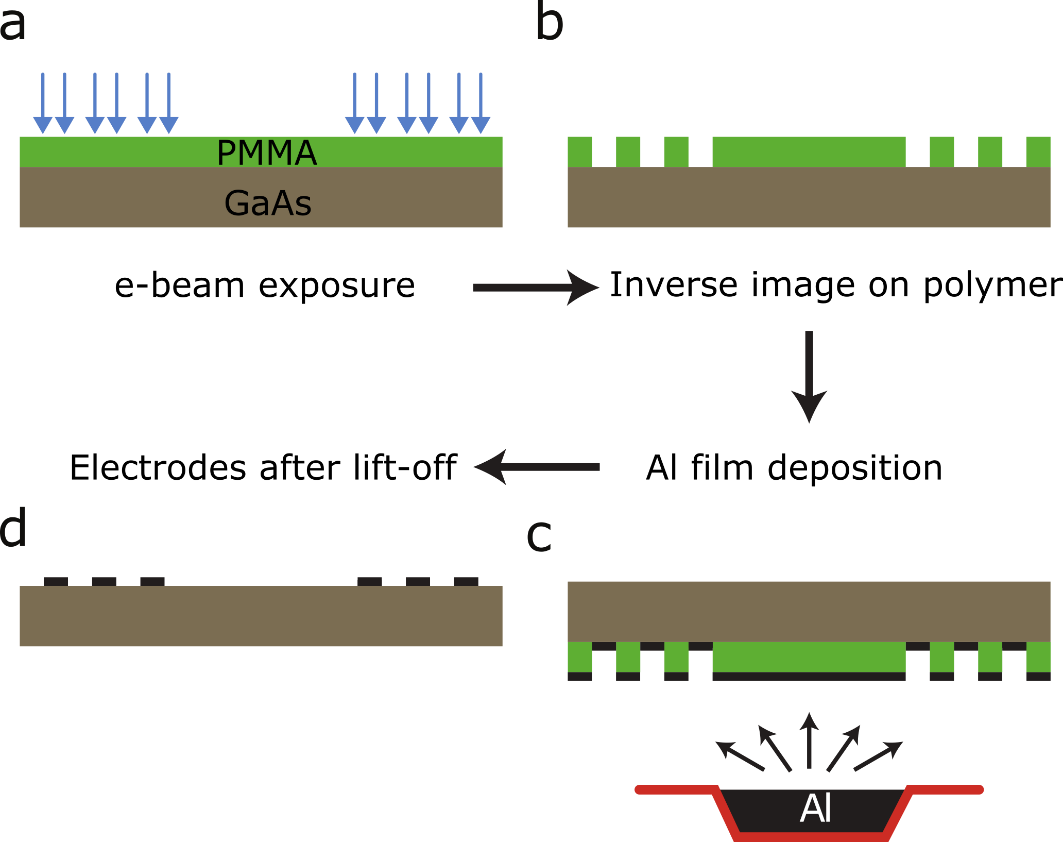


Figure S3: SAW device fabrication process. a. Drawing pattern on a PMMA-coated GaAs chip using electron-beam lithography. b. Inverse image of pattern after development of the polymer in a 3:1 mixture of MIBK and IPA. c. Al film deposition in a UHV e-beam evaporation system. d. Al electrodes on GaAs after lift-off.

The SAW resonators are fabricated on GaAs substrates via a standard e-beam lithography process (Fig. S3). First, a double-side-polished GaAs chip is coated with ∼ 500 nm thick PMMA polymer. The reflector profiles are written onto the polymer with an electron-beam lithography tool (Fig. S3a). In the subsequent step, the polymer broken by e-beam exposure is washed away, resulting in a negative image of the pattern (Fig. S3b). Next, a 200-nm thick Al film is deposited on the chip in an ultra-high vacuum e-beam evaporation system (Fig. S3c). Finally, the chip is removed from the chamber and submerged in a hot acetone bath, which removes PMMA and metal film from unwanted areas, leaving behind Al electrodes (Fig. S3d).

**S5. Optomechanical Spectroscopy**

This Section presents additional details for the experimental spectroscopy apparatus used for measuring the optomechanical response from SAWs (Fig. S4a). A continuous-wave (CW) laser at 1550 nm (the carrier, $\omega_{C}$) is split into two fiber paths to generate the two acoustic drive fields. The first acoustic drive field, drive_1_, is generated by modulating the optical carrier (blue in Fig. S4a) with a null-biased intensity modulator with a frequency $\omega_{1}=2\pi\times11 \mathrm{GHz}$ and then filtering out the upshifted sideband with a fiber-Bragg grating (FBG) (Fig. S4b). The remaining lower frequency optical sideband serves as one of the acoustic drive tones, drive_1_, with frequency $\omega_{d1} ={\omega_{C}-\omega}_{1}$. Similarly, along the second path, the carrier is modulated with a frequency of $\omega_{2}=2\pi\times\left( 11+\Omega\right)\mathrm{GHz}$ and subsequently filtered with an FBG to generate the second acoustic drive, drive_2,_ with frequency $\omega_{d2}=\omega_{c}-\omega_{2}$. The frequency of drive_2_ ($\omega_{d2})$ is tuned continuously so that the difference between the two acoustic drives ($\Omega=\omega_{d1}-\omega_{d2}$) can be varied through targeted acoustic resonance frequencies (Fig.S4b).

Figure S4: a) Experimental apparatus. IM: intensity modulator, FBG: fiber-Bragg grating, Amp: optical amplifier, BD: balanced detector, ESA: electrical spectrum analyzer. b) Acoustic drive field, drive_1_ (drive_2_) are generated by first modulating the optical carrier ($\omega_{c}$) with frequency $\omega_{1}(\omega_{2})$ and then subsequently filtering out the higher frequency sideband. The acoustic wave with frequency $\Omega$ is optically excited by two acoustic drives (drive_1_ at $\omega_{d1}$and drive_2_ at $\omega_{d2}$), and the Stokes ($\omega_{S}$) and anti-Stokes ($\omega_{AS}$) sidebands are generated on either side of the probe ($\omega_{pr}$) with offset, $\Omega$. c) Alignment pads and the acoustic mirrors can be used to align the optical beams to the center of the SAW cavity.


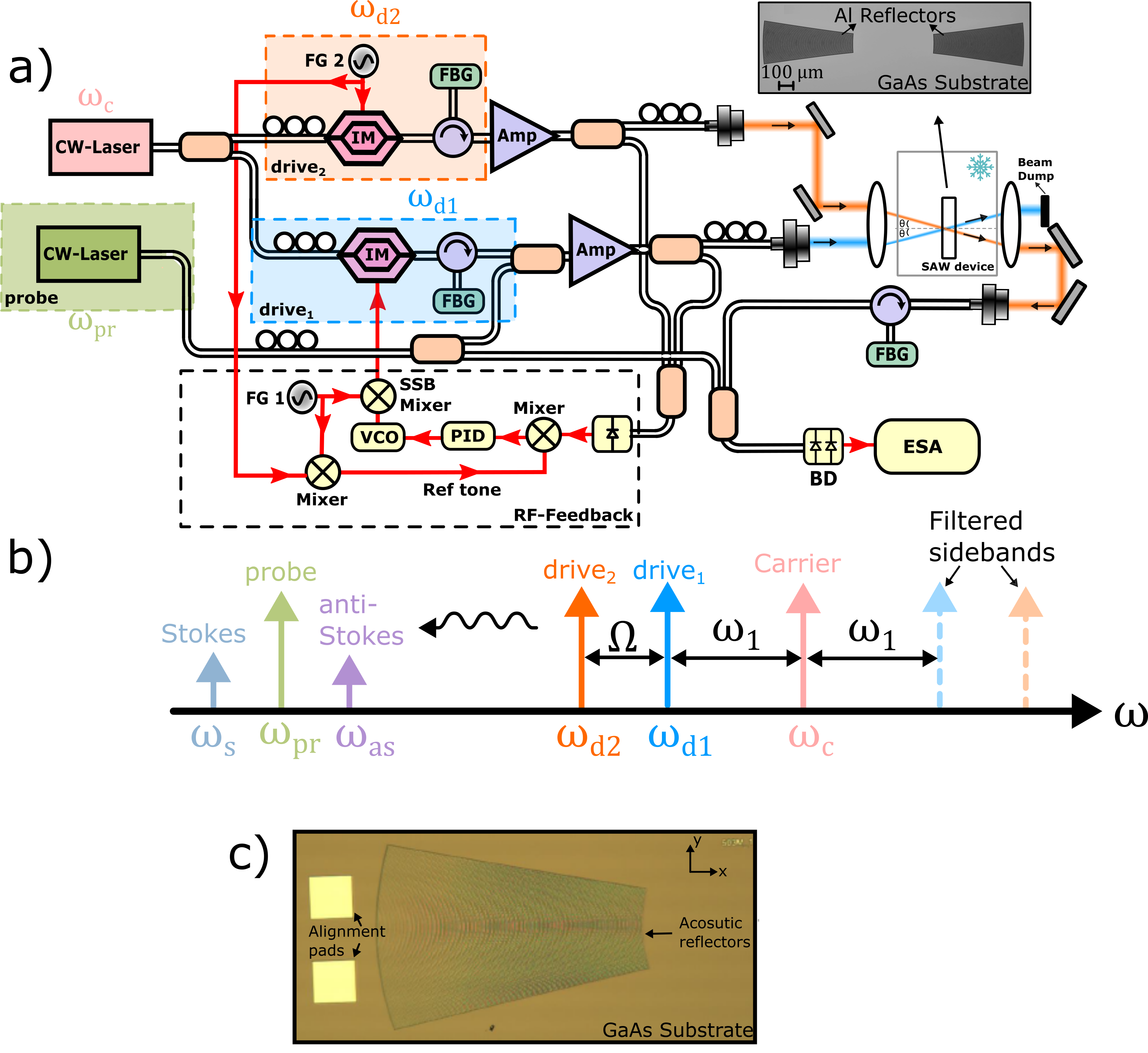


A second CW laser with frequency (colored green in Fig. S4a ), $\omega_{pr}$, is also split into two paths. The optical field along one path is coupled with drive_1_, and serves as the optical probe field to scatter off the driven acoustic field. While the field along the other path serves as the local oscillator and is coupled with the optomechanically scattered field. The three optical fields (drive_1_, drive_2,_ and probe) are amplified by Erbium optical amplifiers to optical powers of about $\sim150-350 mW$ each before impinging on the SAW cavity. Independent polarization controllers are used along each optical field path to ensure that the optical fields are linearly polarized either along TE, or TM.

For optimal optomechanical coupling, the optical beams must be aligned to the center of the SAW cavity. To assist in the process, metallic alignment pads are fabricated outside the SAW cavity with the gap between them coincident with the acoustic cavity axis (Fig. S4d). By monitoring the transmitted power as the optical beam is manually scanned along the y-axis, the alignment pads are located, and the beams are positioned in between them. Similarly, the optical beams can be scanned along the x-axis and the obstruction created by the acoustic mirrors can be used to locate the region in between the acoustic mirrors.

To achieve the desired angle of incidence, the optical fields are collimated and incident off-axis on a focusing aspheric lens. The off-axis displacement is controlled through a linear stage, providing fine control over the incident angle. The dependence of the incident angle on the off-axis displacement is carefully pre-calibrated, as detailed in the following Section. The optical probe, which is collinear to drive_1,_ optomechanically scatters via the driven SAW cavity mode into a signal collinear to drive_2_. This optomechanical signal and drive_2_ are collected with a single-mode collimator and filtered with an FBG filter to reject the excess acoustic drive. The filtered signal is combined with the local oscillator, which was previously split from the probe field, is incident on a balanced detector, and subsequently detected on an electrical spectrum analyzer.

The two acoustic drive tones (drive_1_, drive_2_) can accumulate relative frequency noise as a result of traveling through distinct optical paths with different lengths and components and could limit the measurable linewidths of the optomechanical response. To mitigate the effects of the noise, we implement an optoelectronic feedback loop to lock the relative frequency of the two acoustic drive tones. A small fraction (2 %) of the two acoustic drives is extracted before they are incident on the device under test and mixed on a photodetector. The resulting beat signal is mixed with a reference RF signal generated from the original RF signal generators used to generate the acoustic drives ($\omega_{d1}$ and $\omega_{d2}$). The phase of the resultant signal is then measured on a lock-in amplifier, and a PID produces a correction signal to a voltage-controlled oscillator (VCO) for feedback. This phase-locked loop ensures that the two drive tones are stabilized to a relative frequency of $\sim1 Hz$.

**S6. Calibrating the Optical Angle of Incidence**


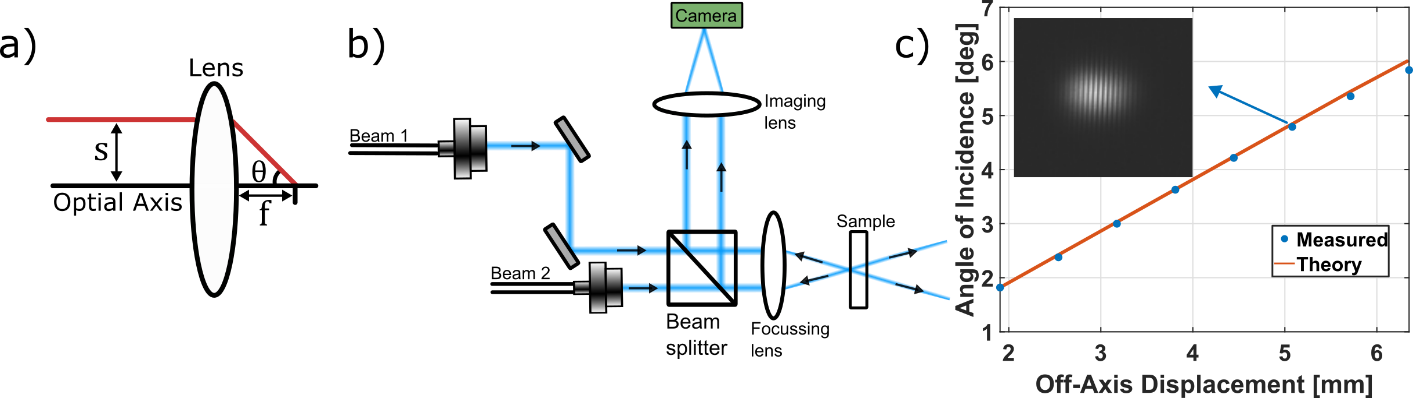


Figure S5: Measurement and calibration of the optical angles of incidence. a) An off-axis ray incident on an aberration-free lens intersects the optical axis at the focal point with an angle given by $\tan\theta=\frac{s}{f}$, b) Experimental apparatus used to measure the angle of incidence by imaging the focal plane, c) Measured angle as a function of off-axis displacement is compared to the ideal lens and displays good agreement. A typical image of the focal plane is inset.

In the paraxial limit, the off-axis optical rays passing through an ideal lens with a focal length of $f$ intersect the optical axis at the focal point with an angle of incidence given by $\theta=\tan^{-1} \left( \frac{s}{f} \right)$, where $s$ is the off-axis displacement (Fig. S5a). Through this relation, the optical angle of incidence can then be controlled through the off-axis displacement on the lens. Note that this relation is approximate and is not valid in general for real lenses with additional aberrations incident with diffracting optical beams.

To validate the relation in the paraxial limit for the components used in practice, an apparatus is developed (Fig. S5b) which images the focus of two intersecting beams for calculation of the angle of incidence as a function of off-axis displacement as determined by the resulting interference pattern (Fig. S5c inset). An optical beam, beam_1,_ is incident along the optical axis of the lens under test, which subsequently focuses on a partially reflective sample placed at the focal plane of the lens. The focused beam is aligned to the surface normal of the sample by maximizing the power of the back-reflected beam. This configuration is assumed to represent $\theta=0^{\circ}$. Next, beam_1_ is laterally displaced by a known distance away from the optical axis. A second beam, beam_2_, is aligned such that the partially reflected beam_1_ maximally couples into the beam_2_ collimator. This alignment ensures the two beams are focused on the same spot on the sample with equal but opposite angles of incidence. A 90:10 beam splitter samples a small portion of the back reflected beams, which are focused using an imaging lens on a near-infrared camera. The image observed on the camera consists of spatial fringes resulting from the interference of beam_1_ and beam_2_ (Fig.S5c inset). The spatial periodicity ($\Lambda$) of the observed fringes can then be used to infer the angle of incidence on the sample through the relation, $\Lambda=\frac{\lambda_{0}}{2\sin\theta}$ [15]. The angle of incidence measured as a function of the off-axis displacement of beam_1_ shows excellent agreement with the prediction in the paraxial limit (Fig. S5c) for an aspheric lens with a focal length of $f=75 mm$. The observed results confirm that geometric aberrations within the lens and other optical components within the system are sufficiently small such that the paraxial approximation is valid.

**S7. Extracting the Optomechanical Coupling Rate from Measurements**

This Section describes how the optomechanical coupling rate, $g_{0}$, is estimated from spectral measurements of the SAW cavity devices. In the system under consideration (Fig. S4b), two optical drive fields with amplitudes $a_{d1}$ and $a_{d2}$, resonantly drive a SAW cavity mode with amplitude $b$. A third optical probe with amplitude, $a_{\mathrm{pr}}$, scatters off the driven phonon mode into newly generated Stokes ($a_{S}$) and anti-Stokes sidebands ($a_{\mathrm{AS}}$). Here two simplifying assumptions are made: first, the system is operated in the small-gain limit, in which pump depletion does not occur and, as a result, incident optical fields $a_{d1}, a_{d2}$and $a_{\mathrm{pr}}$ do not evolve in space. Second, the weak phonon drive generated from the scattered signals ($a_{S}$ and $a_{\mathrm{AS}}$) and the incident probe is neglected. In contrast, in the strong gain limit (e.g. in an optomechanical cavity configuration), both of these assumptions would be invalid and require a more general analysis. Additionally, we assume that for the small angles of incidence (near normal incidence) in this work, the optical beams approximately travel along the z-axis. This analysis could be appropriately modified for larger angles. The equations of motion for the driven cavity phonon amplitude, $b$, and scattered signals, $a_{S}$ and $a_{\mathrm{AS}}$, are given by [3,16,17]

$\frac{\partial b}{\partial t}=-i{(\Omega}_{0}-\Omega)b-\frac{\Gamma}{2}b-i \int g_{0}^{*}a_{d1}^{*}a_{d2}$, ( 86 )

$v_{o}\frac{\partial a_{S}}{\partial z}+\frac{\partial a_{S}}{\partial t}=-ig_{0}^{*}b^{*}a_{\mathrm{pr}}$, ( 87 )

$v_{o}\frac{\partial a_{\mathrm{AS}}}{\partial z}+\frac{\partial a_{\mathrm{AS}}}{\partial t}=-ig_{0}ba_{\mathrm{pr}}^{*}$, ( 88 )

where $v_{o}, \Omega_{0}, \Omega$, and $\Gamma$ refer to the optical group velocity, the resonant phonon frequency, the frequency difference between optical drives, and the acoustic dissipation rate, respectively. The integral in Eq. 86 is performed only over the acoustic decay length, i.e. $l_{a}=\frac{1}{2Re(\eta)q}\sim2 \mu m$ as defined in Section S1 (Eq. 5, 6). Assuming operation in the steady-state ($\partial_{t}=0$) with resonant driving ($\Omega=\Omega_{0}$), the phonon-field amplitude can be expressed as

$b=-i\frac{2}{\Gamma}g_{0}^{*}l_{a}a_{d1}^{*}a_{d2}$. ( 89 )

Inserting Eq. 89 into Eq. 87 and Eq. 88 and assuming $a_{S}\left( 0 \right)=a_{\mathrm{AS}}\left( 0 \right)=0$, gives

$a_{S}=\frac{2{|g}_{0}\left. \right|^{2}l_{a}^{2}}{\Gamma v_{o}}a_{d1}a_{d2}^{*}a_{\mathrm{pr}}$, ( 90 )

$a_{\mathrm{AS}}=\frac{2{|g}_{0}\left. \right|^{2}l_{a}^{2}}{\Gamma v_{o}}a_{d1}^{*}a_{d2}a_{\mathrm{pr}}^{*}$. ( 91 )

The optical power in terms of field amplitude is given by

$P_{i}^{\mathrm{op}}=\hbar\omega_{i}v_{o}{|a}_{i}\left. \right|^{2}$. ( 92 )

Using Eq. 90, 91 and 92, the optomechanically scattered sideband powers can be expressed as

${P_{\mathrm{AS}}=P}_{S}=\frac{\beta^{2}}{\hbar^{2}\omega_{0}^{2}v_{g}^{2}}P_{d1}P_{d2}P_{\mathrm{pr}}$, ( 93 )

where $\beta=\frac{2\left| g_{0} \right|^{2}l_{a}^{2}}{\Gamma v_{o}}$. Here, we also assume $\omega_{as}\approx\omega_{s}\approx\omega_{pr}$, and $\omega_{d1}\approx\omega_{d2}=\omega_{0}$. These optomechanically scattered sidebands are spectrally separated by $\Omega_{0}$ on either side of the incident probe. Assuming a local oscillator with an optical power $P_{\mathrm{LO}}$, the resulting heterodyne beat note oscillating at a frequency $\Omega_{0}$ is given by

$P_{\mathrm{het}}=2\sqrt{P_{\mathrm{LO}}P_{S}}+2\sqrt{P_{\mathrm{LO}}P_{\mathrm{AS}}}=4\sqrt{P_{\mathrm{LO}}P_{\mathrm{AS}}}=\frac{8\left| g_{0} \right|^{2}l_{a}^{2}}{\hbar\omega_{0}{\Gamma v}_{g}^{2}}\sqrt{P_{d1}P_{d2}P_{\mathrm{pr}}P_{\mathrm{LO}}}$. ( 94 )

The coupling rate can then be estimated by inverting equation Eq. 94 as

$g_{0}=\left( \frac{P_{\mathrm{het}}\hbar\omega_{0}\Gamma v_{g}^{2}}{8l_{a}^{2}\sqrt{P_{d1}P_{d2}P_{\mathrm{pr}}P_{\mathrm{LO}}}} \right)^{\frac{1}{2}}$. ( 95 )

**[100]-Oriented Cavities:**

For cavities oriented along the [100] direction, the optical drive tones and probe tone have a free space wavelength of $\lambda_{d1}\approx\lambda_{d2}=1550.05 nm$ and $\lambda_{\mathrm{pr}}=1550.25 nm,$ respectively. The optical fields are incident at an angle of ${7.8}^{\circ}$. Drive_1_, drive_2,_ and probe powers before the sample are$325 mW$, $117 mW$, and $434 mW,$ respectively. The optical reflectivity at the front surface resulting from refractive index mismatch is $29.4 \%$. Since the reflected fields do not contribute to the optomechanical process, the effective powers mediating the optomechanical process are $P_{d1}=228 mW$, $P_{d2}=82 mW$ and $P_{\mathrm{pr}}=304 mW$. All the optical fields are ensured to be TE-polarized (p-polarization) by using a polarizing beam splitter. The local oscillator power is $P_{\mathrm{LO}}=3.7 mW$. The measured heterodyne power is $P_{\mathrm{het}}=0.34 \mu W$. With the numbers, the corresponding experimental estimate for the coupling rate is $\frac{g_{0}}{2\pi}=1.43\times{10}^{3}\mathrm{Hz}$. This agrees well with a theoretically estimated value of $1.73\times{10}^{3}\mathrm{Hz}$. The small mismatch may result from uncalibrated RF losses, polarization mismatch between the optical tones, and errors in the measurement of the optical beam positions and sizes.

**[110]-Oriented Cavities**

For cavities oriented along the [110] direction, optical drive tones, and the probe tone have a free space wavelength of $\lambda_{d1}\approx\lambda_{d2}=1550.05 nm$ and $\lambda_{\mathrm{pr}}=1550.25 nm,$ respectively. The optical fields are incident at an angle of ${7.8}^{\circ}$. Drive_1_, drive_2,_ and probe powers before the sample are$332 mW$, $126 mW$, and $443 mW,$ respectively. The optical reflectivity at the front surface resulting from refractive index mismatch is $29.4 \%$. Since the reflected fields do not contribute to the optomechanical process, the effective powers mediating the optomechanical process are $P_{d1}=232 mW$, $P_{d2}=88.7 mW$ and $P_{\mathrm{pr}}=310 mW$. All the optical fields are ensured to be TE-polarized (p-polarization) by using a polarizing beam splitter. The local oscillator power is $P_{\mathrm{LO}}=3.7 mW$. The measured heterodyne power is $P_{\mathrm{het}}=3.05 nW$. With the numbers, the corresponding experimental estimate for the coupling rate is $\frac{g_{0}}{2\pi}=2.4\times{10}^{3}\mathrm{Hz}$. This agrees well with a theoretically estimated value of $1.8\times{10}^{3}\mathrm{Hz}$.

**S8. Quality Factor vs. Length**

The quality factor of an acoustic cavity (Q) can be expressed as a function of roundtrip loss ($\alpha_{l}$), resonant frequency ($f_{0}$), acoustic velocity ($v_{R}$), cavity length ($L$), and linewidth ($\Delta f$) as [13]

$Q=\frac{f_{0}}{\Delta f}=\frac{{2f}_{0}L}{v_{R}\alpha_{l}}$*.*  ( 96 )

The acoustic round trip loss can be expressed as a sum of the propagating loss and losses occurring in the acoustic mirror. Propagation losses which scale with propagation length, can be characterized through an attenuation coefficient ($\alpha_{P}$) while mirror losses ($\alpha_{M}$) are independent of length. The total loss can then be expressed as

$\alpha_{l}={2(\alpha}_{p}L+\alpha_{M})$. ( 97 )

The factor of two in Eq. 97 is a result of acoustic fields propagating for a total round-trip length of $2L$ while encountering the acoustic mirrors twice, one on each side of the cavity. Inserting Eq. 97 into Eq. 96 gives

$Q= \frac{f_{0}L}{v_{R}} \left( \frac{1}{\alpha_{p}L+\alpha_{M}} \right).$ ( 98 )

If the cavity length or propagation loss is very small (losses are dominated by mirror losses), such that $\alpha_{P}L\ll\alpha_{M}$, Eq. 98 simplifies to

$Q\approx\frac{f_{0}L}{v_{R}\alpha_{M}}$, ( 99 )

where the quality factor has a simple linear dependence on length, as is the case for piezo-inactive cavities. However, further analysis is required for the more typical case of modest (non-zero) propagation loss.

| \| **Parameter** \| **Value (units)** \| **Description** \| \| --- \| --- \| --- \| \| $f_{0}$ \| $475$ MHz \| Acoustic frequency \| \| $v_{R}$ \| $2615$ m/s \| SAW velocity \| \| $\alpha_{p}$ \| ${10}^{-5}-{10}^{-4} \mu m^{-1}$ \| Propagation loss \| \| $\alpha_{M}$ \| $0.9$ \| Mirror loss \| | \| **Parameter** \| **Value (units)** \| **Description** \| \| --- \| --- \| --- \| \| $f_{0}$ \| $510$ MHz \| Acoustic frequency \| \| $v_{R}$ \| $2880$ m/s \| SAW velocity \| \| $\alpha_{p}$ \| ${8\times10}^{-3} \mu m^{-1}$ \| Propagation loss \| \| $\alpha_{M}$ \| $11.2$ \| Mirror loss \| |
| --- | --- | --- | --- | --- | --- | --- | --- | --- | --- | --- | --- | --- | --- | --- | --- | --- | --- | --- | --- | --- | --- | --- | --- | --- | --- | --- | --- | --- | --- | --- | --- |

Table S4: Estimated loss parameters ($\alpha_{p}, \alpha_{M}$) for piezo-inactive (left) and piezo-active (right) cavities.

The Q vs. L measurements are numerically fit to the full theoretical model (Eq. 98) resulting in the fitting values listed in Table. S4 and Fig. 4a-b in the main text. The loss parameters extracted by the numerical fitting procedure for piezo-inactive (Table. S4 left) and piezo-active (Table. S4 right) suggest that in both cavities the mirror loss dominates propagation loss (by ~100x for the piezo-inactive cavities and by ~4x for the piezo-active cavities for cavity length $L=370 \mu m$) and that the propagation and mirror losses within the piezo-cavity are approximately >100x ($\alpha_{p,p}/\alpha_{p,np}$) and 10x ($\alpha_{M,p}/\alpha_{M,np}$) larger, respectively, than those in piezo-inactive cavities. The larger propagation losses in the piezo-active cavities correspond to the reduced slope of Q vs. L. While the larger mirror losses are consistent with the presence of ohmic losses along in the piezo-active cavities, the source for the larger propagation losses in the piezo-active cavities is less clear. One distinguishing feature of [110]-oriented devices is that SAWs along this direction are known as leaky because they intrinsically have a small wavevector component pointing into the bulk of the substrate. Leaking loss is zero along the [110]-direction and is non-zero for directions away from it [18,19]. Since the cavities investigated in this work support Gaussian modes with a range of wavevectors, the intrinsic propagation losses could also be higher. Large differences in intrinsic losses of leaky vs. non-leaky SAWs can be observed in other substrates as well, for e.g. LiN [20,21]. Finally, we note that, in this analysis, it is assumed that mirror losses ${(\alpha}_{M})$ are independent of cavity length. This assumption could fail if the unique shape of the metallic reflectors which constitute the acoustic mirrors affect the ohmic losses within the mirrors due to different displacement distributions within different cavities.

**S9. Absorption-Mediated Optomechanical Effects**

In addition to optomechanical interactions enabled by nonlinear optical forces (photoelastic and radiation pressure), the devices investigated in this work can also have optomechanical interactions mediated by absorption within the metallic reflectors. The two drive fields separated by a resonant cavity frequency excite the corresponding SAW mode when absorbed by the acoustic reflectors, due to thermo-elastic expansion. Subsequently, the optical probe field scatters off the excited SAW cavity mode to produce an optomechanical response analogous to in the parametric case [22,23]. This type of process does not require phase-matching of the optical drives to generate the acoustic fields since the acoustic fields are driven by time-modulated absorptive effects.

The absorptive interaction is accompanied by thermal effects which can be used to discriminate absorptive interactions from the parametric interactions of primary interest to this study. The optomechanical response of the [100]-oriented devices is measured as a function of incident optical power when the optical beams are in the center of the cavity (parametric case) (Fig. S6a), and when the optical fields have significant overlap with the surrounding metallic reflectors (absorption-mediated case) (Fig. S6b). For the case where parametric interactions dominate the optomechanical response (Fig. S6a), negligible power-dependent effects are observed (Fig. S6c-S6d). In stark contrast, the resonant frequency and the quality factor vary significantly as a function of incident power for the absorption-mediated case (Fig. S6c-d). The observed changes suggest that excess optical absorption in the metal strips modifies the characteristics of the resonant mode, consistent with spurious heating of the substrate and associated changes in local elastic properties of the SAW cavity. For example, the decrease in the resonant acoustic frequency as a function of the incident power is consistent with a decrease in the effective SAW velocity resulting from the increase in the effective temperature of the substrate which is typical for most crystalline substrates [24]. Similarly, the decrease in acoustic quality factor is also qualitatively explained by an increase in the effective temperature of the SAW substrate. Both trends (frequency and Q-factor) are consistent, moreover, with temperature-dependent measurements from Fig. 4c-d of the main text. Quantitatively, absorption-mediated processes are difficult to characterize precisely, due to the limitations of the current experimental apparatus. Additionally, the strength of the absorptive scattering is observed to be a function of the position of the optical beams with respect to the acoustic mirror, which is challenging to accurately measure. Despite these limitations, by measuring the power of the scattered sideband for absorptive scattering for a fixed optical drive power, a lower bound (other positions on the mirror could possibly yield stronger absorptive effects) on the coupling strength of the absorptively mediated optomechanical process can be estimated. The Brillouin coupling coefficient of the absorptive process ($G_{a}$) is found to be larger than that of the parametric process ($G_{p}$) by at least 10x, i.e. $\frac{G_{a}}{G_{p}}>10$.


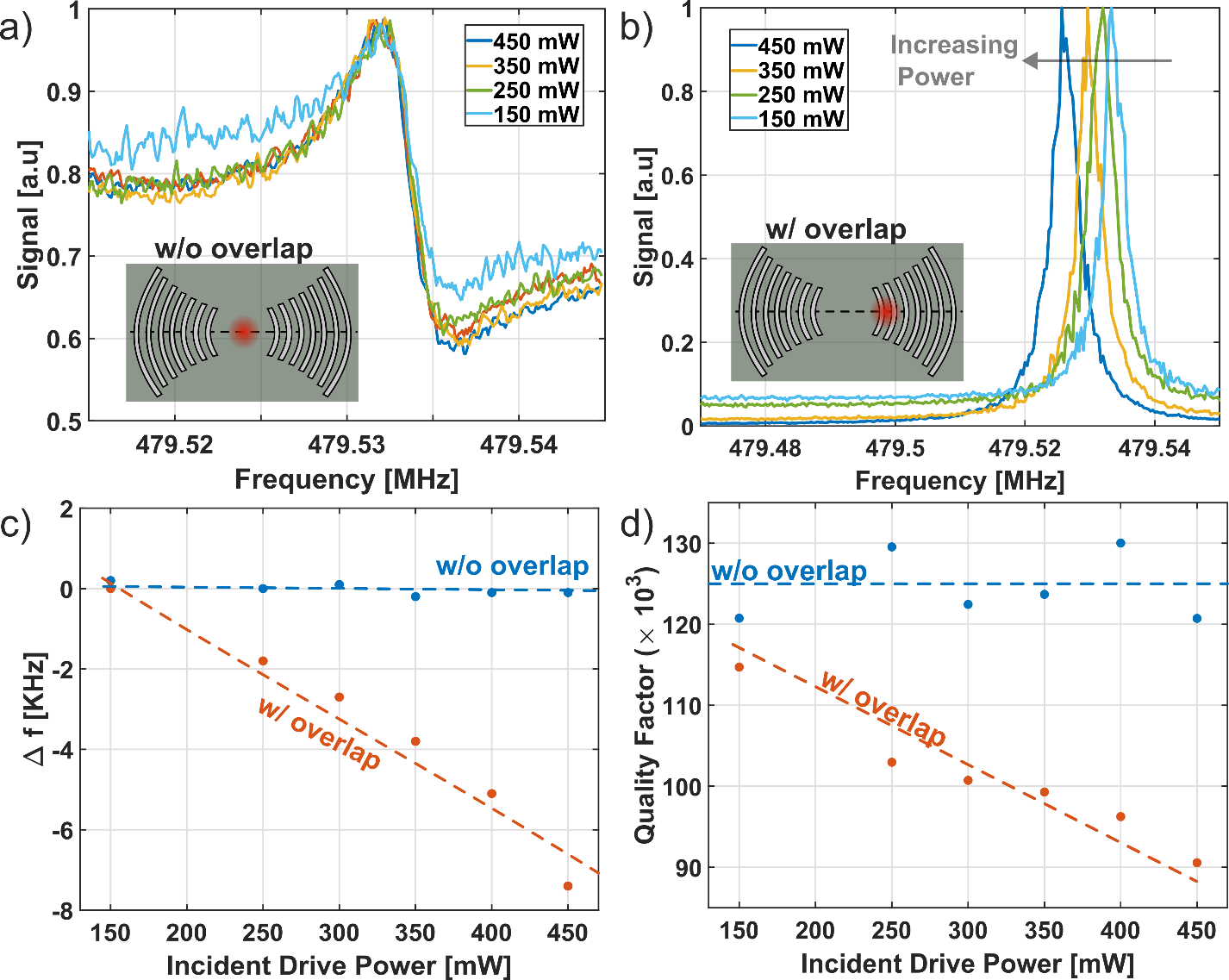


Figure S6. Absorption-mediated optomechanical processes. a) Optomechanical response as a function of incident drive power when the optical fields have minimal overlap with the metallic reflectors (beam illustration inset). b) Optomechanical response as a function of incident optical power when the optical fields overlap with the metallic reflectors (beam illustration inset). c) Change in the resonant frequency ($\Delta f$) of the observed cavity mode as a function of incident optical drive power for the case with (orange) and without (blue) overlap with the reflectors. $\Delta f=0$ is defined at an incident drive power of 150 mW. d) Acoustic quality factor as a function of incident optical power with (orange) and without (blue) optical overlap with the metallic reflectors.

As demonstrated in previous works, such absorptive interactions could be employed for various classical applications, including optical signal processing [22,23]. However, because they are incoherent, absorption-mediated effects would generally be undesirable for applications requiring coherent interactions, including quantum control, transduction, and sensing. Additionally, spurious heating resulting from absorption could prevent the robust ground-state operation of quantum systems such as qubits. These parasitic thermal effects are minimized for devices investigated in this work by ensuring the mirror separation is much larger than the incident optical beam waist. For example, for devices employed in this work with an approximate mirror separation of $500 \mu m$ and an optical beam of waist diameter of $60 \mu m$, the fraction of optical power spatially overlapping with the acoustic mirrors is vanishingly small (e.g. $1-\mathrm{erf} \left( \frac{250 \mu m}{30 \mu m} \right)$). Alternatively, any residual thermal effects can be eliminated by replacing the metallic stripe reflectors with etched grooves for SAW confinement [14,25,26].

**S10. SAW-Mediated Cavity-Optomechanical Devices**

Here we consider a specific variation of a SAW-mediated cavity optomechanical system. We assume a SAW cavity on [100]-cut GaAs optimized for an acoustic wavelength (frequency) of $\lambda_{a}=700 \mathrm{nm}$ ($\Omega_{0}\sim4 GHz$) with a Gaussian waist size of $w_{0}=2\lambda_{a}$ and a cavity length of $L_{\mathrm{eff}}\sim30\lambda_{a}$. This SAW cavity can be phase-matched to $\lambda_{o}=1 \mu m$ optical fields incident at $\theta={45}^{\circ}$. Assuming the fields are TM polarized, Eq. 78 can be used to estimate a traveling-wave coupling rate of $g_{0} \sim2\pi\times400 kHz$. This coupling rate is approximately 250x that of $500 \mathrm{MHz}$ cavities measured in this study. This large enhancement is a result of the acoustic mode volume scaling with the acoustic wavelength (e.g. see Eq. 39 and Eq. 42). For a cavity-optomechanical system, an optical cavity is required that is also compatible with SAW cavities. For this we consider cavity designs based on coated fiber-optic mirrors such as those used for membrane-type cavity-optomechanical devices as well as atomic systems [27,28]. The ability to miniaturize these optical cavities is ideal for obtaining small optical mode volumes and the resultant large cavity-optomechanical coupling strengths. While current fiber cavities are designed for normal incidence operation which are not directly compatible with SAW systems requiring finite angles of incidence for phase-matching, straightforward modifications of existing geometries (e.g. with bow-tie style cavities), could make them compatible for coupling to the SAW cavities described in this study. Assuming conservative optical cavity lengths of 100 microns ($l_{opt}\sim100 \mu m$) and a conservative optical finesse of $\mathcal{F=}{10}^{4}$ (these could be as large as ${10}^{6}$), Eq. 8 can be used to estimate the cavity optomechanical coupling rate as

$g_{0}^{c}=g_{0}\left( \frac{l_{a}}{l_{opt}} \right)\approx2\pi\times550 Hz$. ( 100 )

As described in Section S1, the cavity coupling rate is reduced from the travelling-wave coupling rate ($g_{0}\approx2\pi\times400 kHz$) by the ratio of the short acoustic mode size ($\sim0.5 \mu m$) to the longer optical cavity ($\sim100 \mu m$) size. On the other hand, for loaded-cavity operation, this system can handle very high powers (see Section S13) enabling large intracavity photon numbers ($n_{c}>{10}^{9}$), as is typical for bulk cavity optomechanical systems [29]. The optically loaded cavity optomechanical cooperativity is given by [4]

$C_{\mathrm{om}}=\frac{4g_{0}^{2}n_{c}}{\Gamma\kappa}$, ( 101 )

where $\Gamma$ and $\kappa$ refer to the acoustic and the optical cavity decay rates, respectively. Assuming the experimentally observed quality factor $Q\approx{10}^{5}$ and $g_{0}^{c}\approx2\pi\times550 \mathrm{Hz}$, the cavity optomechanical cooperativity is estimated as

$C_{\mathrm{om}}\approx250.$ ( 102 )

This platform therefore provides the high-power capability of bulk optomechanical systems [5,30], but also offers large coupling rates, a small footprint, and simple integrability to quantum systems and sensing devices. While in general, the quality factor of SAW cavities can degrade as the frequency increases to several GHz [31], previous electromechanical systems have demonstrated intrinsic quality factors exceeding ${10}^{5}$ at $\sim4 \mathrm{GHz}$  [32,33] which should be accessible in the present device designs with further optimization. Moreover, additional increases may be achieved without the need for electrodes and with optimized high frequency Gaussian SAW cavities (e.g. with an optimized mirror geometry based on etched grooves). Since this form of optomechanical interaction can be realized on nearly any substrate and crystallographic direction, regardless of piezoelectricity, the material, and axis can further be chosen to minimize loss.

**S11. Additional Experimental Data**

Here additional experimental data is presented for the [100]-oriented device on [100]-cut GaAs (Fig. S7) related to the strong resonance measured and featured in Fig. 2a-b. As expected for an optomechanical interaction, when the one of the acoustic drives is turned off, no response is observed (green trace, Fig. S7a). An optomechanical response is also not observed when the drives are orthogonally polarized with respect to each other (purple trace Fig. S7a) or the LO is polarized orthogonally (yellow trace, Fig. S7a) to the incident probe optical field. This is consistent with cross-polarized SAW interactions being forbidden within cubic crystalline materials (e.g., Eq. 61). The TM-TM scattering trace is also measured (Fig. S7b), yielding an estimated quality factor of $120,000$ and a scattering strength approximately equal to TE-TE scattering, as expected. Together, these results, in agreement with theoretical predictions, thoroughly support that the measured resonant responses are the result of optomechanical processes.

Figure S7: Additional optomechanical measurement data for the device presented in Fig. 2a-b. a) Additional TE-TE scattering data including when the acoustic drives are turned off (green), the two acoustic drives are orthogonally polarized with respect to each other (purple) and when LO is orthogonally polarized (yellow) with respect to the incident probe. The TE-TE scattering response is also shown for reference (blue). b) Optomechanical response when the all the fields are TM polarized.


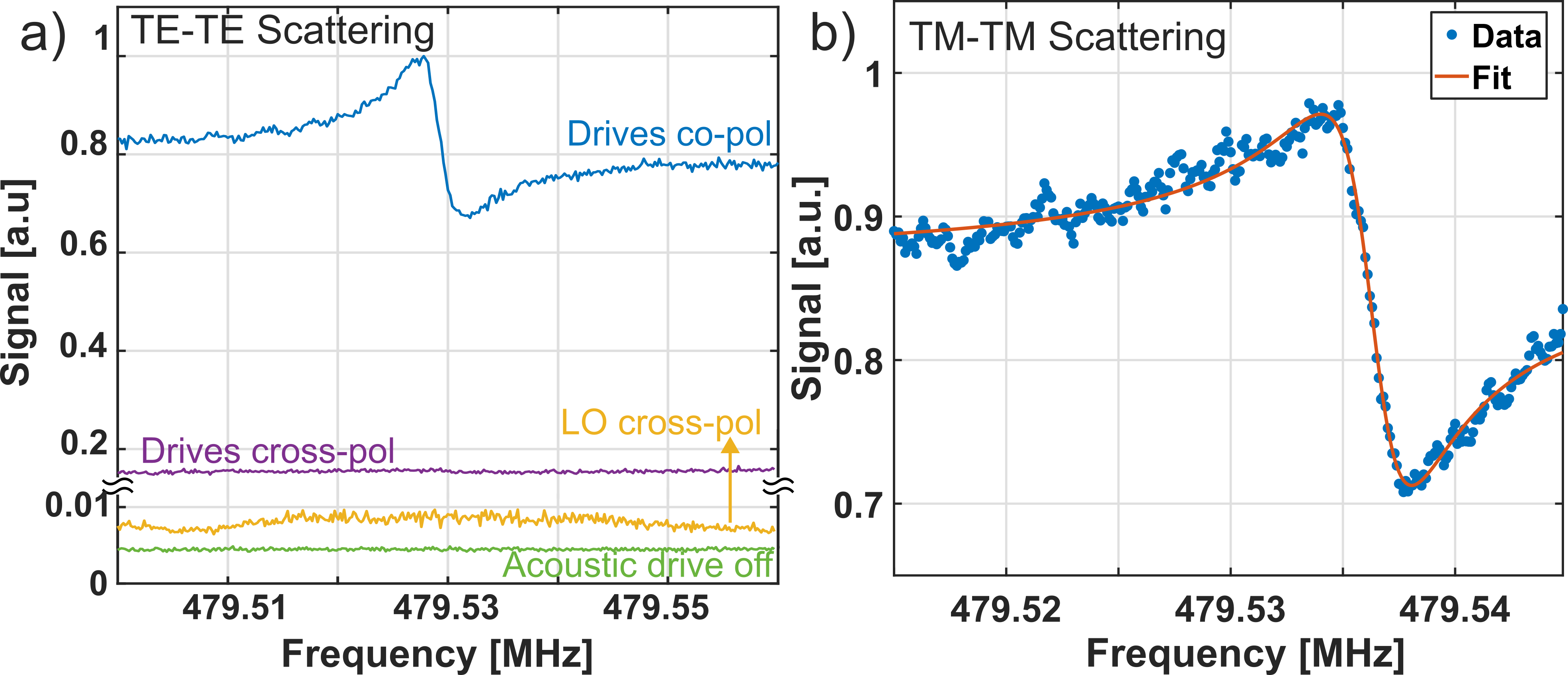


**S12. Theoretical Analysis of the Measured Optomechanical Response**

The procedure for extracting relevant resonance parameters from an experimentally measured optomechanical frequency response is described here. Optomechanical resonances measured through a drive-probe technique, as in this work, are a result of coherent interference with four-wave-mixing-like processes mediated by other physical mechanisms including $\chi^{(3)}$ and free-carriers within the bulk of the substrate [34–36] . These background processes exhibit minimal frequency dependence over the width of the acoustic resonances and therefore are modeled as a coherent frequency independent background. The scattered Stokes (or anti-Stokes) power as a function of frequency from a Fano-like resonance can be expressed as [35]

$P_{s}=P_{nl} \left| e^{i\phi}+\frac{D_{m}\Omega_{m}/\left( 2Q_{m} \right)}{\Omega_{m}-\Omega-i\Omega_{m}/\left( 2Q_{m} \right)} \right|^{2}+P_{bg}$, ( 103 )

where $\Omega_{m}$ and $Q_{m}$ refer to the resonant frequency and quality factor of an acoustic resonance with index $m, P_{nl}$ is the power from frequency independent nonlinear processes,$D_{m}$ is the relative strength between the Brillouin and frequency independent interactions, and $\phi_{m}$ is the relative phase between the resonant Brillouin interaction and the coherent non-resonant background signal (e.g., from the Kerr nonlinearity), and$P_{bg}$ accounts for residual incoherent background noise. The experimentally measured scattered power as a function of the drive frequency,$\Omega$, is given by $P_{s}^{ex}(\Omega)$. This resonance data can be fit to give parameters $p_{m}=\{D_{m},\phi_{m},\Omega_{m},Q_{m},P_{nl},P_{bg}\}$ such that

$P_{s}^{ex}\left( \Omega\right)=P_{s}\left( \Omega,p_{m} \right)$. ( 104 )

It is worth noting that the optomechanical resonances observed within piezo-inactive SAW cavities exhibit a Fano-like line shape typical of optomechanical experiments, whereas resonances observed in piezo-active cavities are nearly Lorentzian. The shape of a Fano-like feature depends on the relative phase between the optomechanical response and the nonresonant background arising from processes including Kerr nonlinear processes. This phase relationship with the non-resonant background between the two cavities could result from their unique frequencies and coupling strengths. Previous works investigating optomechanical processes within systems with complex elastic structures such as photonic crystal fibers [37] have observed stark changes in resonance line shapes within the same device suggesting complex dependencies underlying the interference of optomechanical processes with coherent background processes. This is an important area for future investigation.

**S13. Optical Power Handling Capabilities of SAW-based Optomechanical Systems**

SAW-based optomechanical systems can support high powers owing to the desirably small optical absorption and scattering from the planar crystalline substrate. For optical scattering, the fraction of optical power scattered from a material surface characterized by an RMS (root-mean squared) surface roughness, $\sigma$, can be determined through a standard scattering analysis as $S=S_{F}+S_{B}=R_{0}\left( \frac{4\pi\sigma}{\lambda} \right)^{2}+T_{0}\left( \frac{2\pi\sigma}{\lambda}\left( n-1 \right) \right)^{2}$  [38–41]. Here the total scattering coefficient ($S$), is expressed as a sum of back scattering ($S_{B}$) and forward scattering ($S_{F}$) coefficients. $R_{0}, T_{0}, \theta_{i}$, $\lambda$ and $n$ are the scatter-free reflection coefficient, scatter-free transmission coefficient, the angle of incidence, the optical wavelength, and the refractive index, respectively. The commercially polished GaAs substrates used in this work have surface roughness of $\sigma=0.4 nm$ [42] (this could be as low as $0.1 nm$ [43]). The fraction of scattered optical power for an incident wavelength of $\lambda=1.5 \mu m$ is then $S\approx4\times{10}^{-5} (\sim0.005 \%)$ (and as low as S=$1\times{10}^{-6}$ for $\sigma=0.1 nm$). The low scattering estimate is primarily a result of the large optical wavelength of operation (1550 nm) and low surface RMS roughness (0.4 nm). These scattering estimates are consistent with experimental works measuring scattering from smooth optical components, such as lenses, which are typically of the order of $\sim{10}^{-5}$ (i.e. tens of ppm) [44,45]. For a more conservative estimate, we assume a scattering coefficient an order of magnitude larger than the numerical estimates ($S\sim4\times{10}^{-4}$), to account for additional scattering within the bulk of the material or other exceptionally strong, but spurious scattering events. We also assume that all the scattered power is subsequently absorbed within the chamber and must be cooled. To estimate absorption, the system is first assumed to be well coupled to the thermal bath because of the large (e.g. 4mm x 4mm) substrate contact area (this is in contrast to other nanoscale/microscale optomechanical devices that are suspended or with holes and other features may have a reduced thermal link in a high vacuum environment). The absorbed optical power ($P_{a}$) can be expressed as $P_{a}=\alpha L_{s}P_{in}$ for absorption coefficient, $\alpha$, substrate length, $L_{s}$, and intracavity optical power, $P_{in}$. For crystalline GaAs the absorption coefficient at $1.55 \mu m$ and temperature $<4K$ is given by $\alpha=0.01 cm^{-1}$  [46], and the substrate length used in this work is $L_{s}=300 \mu m$. To ensure that the system stays at the desired temperature, the heating from absorption and the scattered optical power should be less than the cooling power of the cryostat, ($P_{c}$), i.e. $P_{c}\geq P_{s}+P_{a}=(S+\alpha L_{s})P_{in}$ or $P_{in}\leq\frac{P_{c}}{(S+\alpha L_{s})}$. Assuming a cooling power of $P_{c}=10 \mu W at 10 mK$, $P_{c}=1000 \mu W$ at $T=100 mK$ and $P_{c}=300 mW$ at $T=1K$  [47,48] the maximum allowable optical powers to prevent sample heating are $P_{in}\approx13 mW$ $at T=10 mK$, $P_{in}\approx1 W$ $at T=100 mK$ and $P_{in}\approx100 W$ $\mathrm{at} T=1 K$. With the conservative assumptions described above, the contribution due to scattering is approximately the same as that from absorption (i.e. $S\approx\alpha L_{s})$. These estimates are consistent with membrane-type optomechanical systems showing negligible effect of laser heating with $\sim100 mW$ circulating powers at $T\sim40 mK$ [49,50]. In addition, a recent demonstration with $\sim1 W$ circulating power shows no effects of absorption on the phonon occupation of bulk acoustic resonators in their quantum ground state at $T\sim100 mK$ [51] for comparable resonator sizes $(\sim mm-cm$).

Note that for scattering, other device structures, such as acoustic mirrors and lenses, as well as the crystal’s back surface can induce additional scattering, which would need to be sufficiently suppressed. Straight-forward improvements including anti-reflective coatings and larger mirror separations to decrease spatial overlap with acoustic mirrors will assist in mitigating excess scattering. For absorption, materials with lower intrinsic optical losses such as quartz and diamond can be expected to support larger operational powers. Larger powers can also be achieved by reducing the substrate thickness. Since acoustic modes penetrate only a few wavelengths into the bulk, this approach would have a negligible effect on optomechanical dynamics. In contrast to the crystalline surface acoustic wave cavities presented here, conventional micro- and nano-optomechanical systems with lithographically defined nanostructures (holes, suspended structures, etc.), can have much poorer surface quality (e.g. $\sigma\approx5 nm$ [52,53]) resulting in higher scattering losses, in addition to thermal effects resulting from optical absorption.

**References**

1. M. J. A. Schuetz, E. M. Kessler, G. Giedke, L. M. K. Vandersypen, M. D. Lukin, and J. I. Cirac, "Universal quantum transducers based on surface acoustic waves," Phys. Rev. X **5**, (2015).

2. D. Rover and E. Dieulesaint, "Rayleigh wave velocity and displacement in orthorhombic, tetragonal, hexagonal, and cubic crystals," J. Acoust. Soc. Am. **76**, 1438–1444 (1984).

3. G. S. Wiederhecker, P. Dainese, and T. P. M. Alegre, "Brillouin optomechanics in nanophotonic structures," APL Photonics **4**, (2019).

4. M. Aspelmeyer, T. J. Kippenberg, and F. Marquardt, "Cavity optomechanics," Rev. Mod. Phys. **86**, 1391–1452 (2014).

5. W. H. Renninger, P. Kharel, R. O. Behunin, and P. T. Rakich, "Bulk crystalline optomechanics," Nat Phys (2018).

6. P. Kharel, R. O. Behunin, W. H. Renninger, and P. T. Rakich, "Noise and dynamics in forward Brillouin interactions," Phys. Rev. A **93**, 063806 (2016).

7. A. Okada, F. Oguro, A. Noguchi, Y. Tabuchi, R. Yamazaki, K. Usami, and Y. Nakamura, "Cavity Enhancement of Anti-Stokes Scattering via Optomechanical Coupling with Surface Acoustic Waves," Phys. Rev. Appl. **10**, 1 (2018).

8. W. Qiu, P. T. Rakich, H. Shin, H. Dong, M. Soljačić, and Z. Wang, "Stimulated Brillouin scattering in nanoscale silicon step-index waveguides: a general framework of selection rules and calculating SBS gain," Opt. Express **21**, 31402 (2013).

9. H. Shin, W. Qiu, R. Jarecki, J. A. Cox, R. H. Olsson, A. Starbuck, Z. Wang, and P. T. Rakich, "Tailorable stimulated Brillouin scattering in nanoscale silicon waveguides," Nat. Commun. **4**, (2013).

10. P. T. Rakich, P. Davids, and Z. Wang, "Tailoring optical forces in waveguides through radiation pressure and electrostrictive forces," Opt. Express **18**, 14439 (2010).

11. D. Rover and E. Dieulesaint, "Rayleigh wave velocity and displacement in orthorhombic, tetragonal, hexagonal, and cubic crystals," J. Acoust. Soc. Am. **76**, 1438 (1998).

12. M. E. Msall and P. V. Santos, "Focusing Surface-Acoustic-Wave Microcavities on GaAs," Phys. Rev. Appl. **13**, 014037 (2020).

13. R. A. Decrescent, Z. Wang, P. Imany, R. C. Boutelle, C. A. McDonald, T. Autry, J. D. Teufel, S. W. Nam, R. P. Mirin, and K. L. Silverman, "Large Single-Phonon Optomechanical Coupling between Quantum Dots and Tightly Confined Surface Acoustic Waves in the Quantum Regime," Phys. Rev. Appl. **18**, 034067 (2022).

14. P. Imany, P. Imany, P. Imany, Z. Wang, Z. Wang, R. A. DeCrescent, R. C. Boutelle, C. A. McDonald, C. A. McDonald, T. Autry, S. Berweger, P. Kabos, S. W. Nam, R. P. Mirin, K. L. Silverman, and K. L. Silverman, "Quantum phase modulation with acoustic cavities and quantum dots," Opt. Vol. 9, Issue 5, pp. 501-504 **9**, 501–504 (2022).

15. G. M. Burrowg and T. K. Gaylord, "Multi-beam interference advances and applications: Nano-electronics, photonic crystals, metamaterials, subwavelength structures, optical trapping, and biomedical structures," Micromachines **2**, 221–257 (2011).

16. W. H. Renninger, P. Kharel, R. O. Behunin, and P. T. Rakich, "Bulk crystalline optomechanics," Nat. Phys. **14**, 601–607 (2018).

17. W. Xu, A. Iyer, L. Jin, S. Y. Set, and W. H. Renninger, "Strong Optomechanical Interactions with Long-lived Fundamental Acoustic Waves," (2022).

18. M. R. Melloch and R. S. Wagers, "Propagation loss of the acoustic pseudosurface wave on (ZXt)45°GaAs," Appl. Phys. Lett. **43**, 1008–1009 (1983).

19. W. D. Hunt and B. J. Hunsinger, "A precise angular spectrum of plane-waves diffraction theory for leaky wave materials," J. Appl. Phys. **64**, 1027–1032 (1988).

20. K. Yamanouchi and M. Takeuchi, "Applications for piezoelectric leaky surface waves," IEEE Symp. Ultrason. **1**, 11–18 (1990).

21. L. Kelly, P. Berini, and X. Bao, "Measuring Velocity, Attenuation, and Reflection in Surface Acoustic Wave Cavities Through Acoustic Fabry-Pérot Spectra," IEEE Trans. Ultrason. Ferroelectr. Freq. Control **69**, 1542–1548 (2022).

22. M. Katzman, M. Katzman, D. Munk, D. Munk, M. Priel, M. Priel, E. Grunwald, E. Grunwald, M. Hen, M. Hen, N. Inbar, M. Feldberg, T. Sharabani, R. Zektzer, G. Bashan, G. Bashan, M. Vofsi, U. Levy, A. Zadok, and A. Zadok, "Surface acoustic microwave photonic filters in standard silicon-on-insulator," Opt. Vol. 8, Issue 5, pp. 697-707 **8**, 697–707 (2021).

23. D. Munk, M. Katzman, M. Hen, M. Priel, M. Feldberg, T. Sharabani, S. Levy, A. Bergman, and A. Zadok, "Surface acoustic wave photonic devices in silicon on insulator," Nat. Commun. 2019 101 **10**, 1–9 (2019).

24. R. Tarumi, K. Nakamura, H. Ogi, and M. Hirao, "Complete set of elastic and piezoelectric coefficients of α -quartz at low temperatures," J. Appl. Phys. **102**, (2007).

25. L. Shao, S. Maity, L. Zheng, L. Wu, A. Shams-Ansari, Y. I. Sohn, E. Puma, M. N. Gadalla, M. Zhang, C. Wang, E. Hu, K. Lai, and M. Lončar, "Phononic Band Structure Engineering for High- Q Gigahertz Surface Acoustic Wave Resonators on Lithium Niobate," Phys. Rev. Appl. **12**, 014022 (2019).

26. P. Imany, Z. Wang, C. A. McDonald, T. Autry, S. Berweger, R. C. Boutelle, P. Kabos, R. P. Mirin, and K. L. Silverman, "Etched-groove focusing GaAs surface acoustic wave cavities for enhanced coupling to quantum emitters," Conf. Lasers Electro-Optics (2021), Pap. STh1D.7 STh1D.7 (2021).

27. D. Hunger, T. Steinmetz, Y. Colombe, C. Deutsch, T. W. Hänsch, and J. Reichel, "A fiber Fabry–Perot cavity with high finesse," New J. Phys. **12**, 065038 (2010).

28. N. E. Flowers-Jacobs, S. W. Hoch, J. C. Sankey, A. Kashkanova, A. M. Jayich, C. Deutsch, J. Reichel, and J. G. E. Harris, "Fiber-cavity-based optomechanical device," Appl. Phys. Lett. **101**, 221109 (2012).

29. P. Kharel, Y. Chu, E. A. Kittlaus, N. T. Otterstrom, S. Gertler, and P. T. Rakich, "Multimode strong coupling in cavity optomechanics," (2018).

30. P. Kharel, Y. Chu, D. Mason, E. A. Kittlaus, N. T. Otterstrom, S. Gertler, and P. T. Rakich, "Multimode Strong Coupling in Cavity Optomechanics," Phys. Rev. Appl. **18**, 024054 (2022).

31. R. Manenti, M. J. Peterer, A. Nersisyan, E. B. Magnusson, A. Patterson, and P. J. Leek, "Surface acoustic wave resonators in the quantum regime," Phys. Rev. B **93**, 041411 (2016).

32. G. Andersson, S. W. Jolin, M. Scigliuzzo, R. Borgani, M. O. Tholén, J. C. Rivera Hernández, V. Shumeiko, D. B. Haviland, and P. Delsing, "Squeezing and Multimode Entanglement of Surface Acoustic Wave Phonons," PRX Quantum **3**, 010312 (2022).

33. G. Andersson, A. L. O. Bilobran, M. Scigliuzzo, M. M. de Lima, J. H. Cole, and P. Delsing, "Acoustic spectral hole-burning in a two-level system ensemble," npj Quantum Inf. 2021 71 **7**, 1–5 (2021).

34. H. Shin, W. Qiu, R. Jarecki, J. A. Cox, R. H. Olsson, A. Starbuck, Z. Wang, and P. T. Rakich, "Tailorable stimulated Brillouin scattering in nanoscale silicon waveguides," Nat. Commun. 2013 41 **4**, 1–10 (2013).

35. E. A. Kittlaus, H. Shin, and P. T. Rakich, "Large Brillouin amplification in silicon," Nat. Photonics **10**, 463–467 (2016).

36. A. Iyer, W. Xu, J. E. Antonio-Lopez, R. A. Correa, and W. H. Renninger, "Ultra-low Brillouin scattering in anti-resonant hollow-core fibers," APL Photonics **5**, (2020).

37. W. H. Renninger, H. Shin, R. O. Behunin, P. Kharel, E. A. Kittlaus, and P. T. Rakich, "Forward Brillouin scattering in hollow-core photonic bandgap fibers," New J. Phys. **18**, 025008 (2016).

38. T. V Vorburger, E. Marx, and T. R. Lettieri, "Regimes of Surface Roughness Measurable With Light Scattering," Appl. Opt. **32**, 3401–3408 (1993).

39. H. ~E. Bennett and J. ~O. Porteus, "Relation Between Surface Roughness and Specular Reflectance at Normal Incidence," J. Opt. Soc. Am. **51**, 123 (1961).

40. P. Beckmann and A. Spizzichino, *The Scattering of Electromagnetic Waves from Rough Surfaces* (1987).

41. S. Schröder, S. Gliech, and A. Duparré, "Measurement system to determine the total and angle-resolved light scattering of optical components in the deep-ultraviolet and vacuum-ultraviolet spectral regions," Appl. Opt. **44**, 6093–6107 (2005).

42. M. Corporation, "No Title," https://www.mtixtl.com/GaAs-Un-100305S2-VGF-1-2-1-1-1.aspx.

43. Edmund optics, "SUPERPOLISHED OPTICS," https://www.edmundoptics.com/knowledge-center/trending-in-optics/superpolished-optics/.

44. E. Collett, *Optical Scattering* (2009).

45. E. M. Capote, A. Gleckl, J. Guerrero, M. Rezac, R. Wright, and J. R. Smith, "Measurements of Optical Scatter Versus Annealing Temperature for Amorphous Ta2O5 and TiO2:Ta2O5 Thin Films," **5**, 7–9 (2020).

46. M. D. Sturge, "Optical Absorption of Gallium Arsenide between 0.6 and 2.75 eV," Phys. Rev. **127**, 768 (1962).

47. Bluefors, "XLDsl, Dilution Refrigerator Measurement System," https://bluefors.com/products/dilution-refrigerator-measurement-systems/xldsl-dilution-refrigerator-measurement-system/.

48. Bluefors, "Cryomech, 1K Cryostat," https://bluefors.com/products/1k-systems/1k-cryostat/.

49. R. W. Peterson, T. P. Purdy, N. S. Kampel, R. W. Andrews, P. L. Yu, K. W. Lehnert, and C. A. Regal, "Laser Cooling of a Micromechanical Membrane to the Quantum Backaction Limit," Phys. Rev. Lett. **116**, 1–6 (2016).

50. E. Planz, X. Xi, T. Capelle, E. C. Langman, and A. Schliesser, "Membrane-in-the-middle optomechanics with a soft-clamped membrane at milliKelvin temperatures," Opt. Express **31**, 41773 (2023).

51. H. M. Doeleman, T. Schatteburg, R. Benevides, S. Vollenweider, D. Macri, and Y. Chu, "Brillouin optomechanics in the quantum ground state," (2023).

52. G. Arregui, R. C. Ng, M. Albrechtsen, S. Stobbe, C. M. Sotomayor-Torres, and P. D. García, "Cavity Optomechanics with Anderson-Localized Optical Modes," Phys. Rev. Lett. **130**, 1–7 (2023).

53. M. Mitchell, D. P. Lake, and P. E. Barclay, "Realizing Q > 300 000 in diamond microdisks for optomechanics via etch optimization," APL Photonics **4**, 0–11 (2019).
